# Supplementary material for: Supporting adolescents’ participation in muscle-strengthening physical activity: protocol for the ‘Resistance Training for Teens’ (RT4T) hybrid type III implementation–effectiveness trial
Source: BMJ Open. 2023 Nov 1;13(11):e075488. doi: 10.1136/bmjopen-2023-075488 (PMC10626834; doi:10.1136/bmjopen-2023-075488)
Supplement: Author's manuscript [file bmjopen-2023-075488.draft_revisions.pdf]

BMJ Open is committed to open peer review. As part of this commitment we make the peer review history of every article we publish publicly available.

When an article is published we post the peer reviewers' comments and the authors' responses online. We also post the versions of the paper that were used during peer review. These are the versions that the peer review comments apply to.

The versions of the paper that follow are the versions that were submitted during the peer review process. They are not the versions of record or the final published versions. They should not be cited or distributed as the published version of this manuscript.

BMJ Open is an open access journal and the full, final, typeset and author-corrected version of record of the manuscript is available on our site with no access controls, subscription charges or pay-per-view fees (http://bmjopen.bmj.com).

If you have any questions on BMJ Open's open peer review process please email info.bmjopen@bmj.com

BMJ Open

Supporting adolescents' participation in muscle-strengthening physical activity: Protocol for the 'Resistance Training for Teens' (RT4T) hybrid type III implementation-effectiveness trial

Journal:	*BMJ Open*
Manuscript ID	bmjopen-2023-075488
Article Type:	Protocol
Date Submitted by the Author:	10-May-2023
Complete List of Authors:	Kelly, Hayden; The University of Newcastle, School of Education<br>Smith, Jordan ; University of Newcastle, School of Education<br>Verdonschot, Angeliek; The University of Newcastle, School of Education<br>Kennedy, Sarah; Western Sydney University, School of Health Sciences<br>Scott, Joseph; University of the Sunshine Coast; Edith Cowan University, School of Education<br>McKay, Heather; The University of British Columbia, Family Practice<br>Nathan, Nicole; Population Health Hunter New England Local Health District; Hunter Medical Research Institute<br>Sutherland, Rachel; Population Health Hunter New England Local Health District; The University of Newcastle, School of Medicine and Public Health<br>Morgan, Philip; University of Newcastle, PRC for Physical Activity and Nutrition<br>Salmon, Jo; Deakin University,<br>Penney, Dawn; Deakin University<br>Boyer, James; NSW Department of Education, School Sport Unit<br>Lloyd, Rhodri S. ; Cardiff Metropolitan University,<br>Oldmeadow, Christopher; Hunter Medical Research Institute<br>Reeves, Penny; University of Newcastle Hunter Medical Research Institute, Health Research Economics; University of Newcastle, School of Medicine and Public Health<br>Pursey, Kirrilly; The University of Newcastle, School of Health<br>Hua, Myna; Western Sydney University Translational Health Research Institute, School of Health Science<br>Longmore, Sarah; Western NSW Local Health District, Health Promotion<br>Norman , Jennifer ; University of Wollongong Illawarra Health and Medical Research Institute; Illawarra Shoalhaven Local Health District, Health Promotion Service<br>Voukelatos, Alexander; University of New South Wales, Population Health Research and Evaluation Hub, Sydney Local Health District<br>Zask, Avigdor; Northern NSW Local Health District, Health Promotion<br>Lubans, David; The University of Newcastle, School of Education
Keywords:	Adolescent, Schools, EDUCATION & TRAINING (see Medical Education & Training)

SCHOLARONE™
Manuscripts

Supporting adolescents' participation in muscle-strengthening physical activity: Protocol for the 'Resistance Training for Teens' (RT4T) hybrid type III implementation-effectiveness trial

Hayden T, Kelly[1], Jordan J, Smith[1,2], Angeliek Verdonschot[1], Sarah G, Kennedy[3], Joseph, Scott[4,5], Heather McKay[6], Nicole Nathan[2,7,8,9], Rachel Sutherland[2,7,8,9], Philip J, Morgan[1], Jo Salmon[10], Dawn Penney[10], James Boyer[11], Rhodri S, Lloyd[12], Christopher Oldmeadow[13], Penny Reeves[13], Kirrilly Pursey[14,15], Myna Hua[3], Sarah Longmore[16], Jennifer Norman[17], Alexander Voukelatos[18], Avigdor Zask[19,20], and David R, Lubans[1,2,21,*]

[1] Centre for Active Living and Learning, School of Education, University of Newcastle, Callaghan, New South Wales, Australia

[2] Hunter Medical Research Institute, New Lambton Heights, New South Wales, Australia

[3]School of Health Science, Translational Health Research Institute, Western Sydney University, Kingswood, Australia

[4] University of the Sunshine Coast, Queensland, Australia

[5] School of Education, Edith Cowan University, Perth, Western Australia, Australia

[6] University of British Columbia, Vancouver, Canada

[7] Hunter New England Population Health, Hunter New England Area Health Service, Newcastle Australia

[8]School of Medicine and Public Health, The University of Newcastle, Newcastle, New South Wales, Australia

[9]National Centre of Implementation Science, Newcastle, New South Wales, Australia

[10] Deakin University, Geelong, Victoria, Australia

[11] NSW Department of Education, Sydney, New South Wales, Australia

[12] Youth Physical Development Centre, Cardiff School of Sport and Health Sciences, Cardiff Metropolitan University, Wales

[13]Hunter Medical Research Institute, College of Health, Medicine and Wellbeing, University of Newcastle, New South Wales, Australia

[14]School of Health Sciences, College of Health, Medicine and Wellbeing, University of Newcastle, Callaghan, New South Wales, Australia

[15]Food and Nutrition Research Group, Hunter Medical Research Institute, New Lambton Heights, New South Wales, Australia

For peer review only

For peer review only - http://bmjopen.bmj.com/site/about/guidelines.xhtml

[16]Health Promotion, Western New South Wales Local Health District, Bathurst, New South Wales, Australia

[17]Health Promotion Service, Illawarra Shoalhaven Local Health District, Warrawong, New South Wales, Australia

[18]Population Health Research and Evaluation Hub, Sydney Local Health District, School of Population Health, University of New South Wales, New South Wales Australia

[19]Health Promotion, Northern NSW Local Health District, Lismore

[20]University Centre of Rural Health (Northern Rivers), School of Public Health, University of Sydney, Lismore, NSW Australia

[21]Faculty of Sport and Health Sciences, University of Jyväskylä, Jyväskylä, Finland

Running title

RT4T implementation-effectiveness trial

***Corresponding author**

Professor David Lubans

College of Human and Social Futures, School of Education, University of Newcastle

ATC 303, Advanced Technology Centre

Callaghan, University Drive, NSW, 2308, Australia

Phone: +612 4921 2049

Email: David.Lubans@newcastle.edu.au

Word count: 4,630 (excluding authorship statement, reference list and table 1)*

Keywords: School, Resistance training, Implementation, Adolescents, Physical literacy

For peer review only - http://bmjopen.bmj.com/site/about/guidelines.xhtml

ABSTRACT

Introduction: In Australia, only 22% of male and 8% of female adolescents meet the muscle-strengthening physical activity guidelines, and few school-based interventions support participation in resistance training (RT). After promising findings from our effectiveness trial, we conducted a state-wide dissemination of the '*Resistance Training for Teens'* (RT4T) intervention from 2015 to 2020. Despite high estimated reach, we found considerable variability in program delivery and teachers reported numerous barriers to implementation. Supporting schools when they first adopt evidence-based programs may strengthen program fidelity, sustainability, and by extension, program impact. However, the most effective implementation support model for RT4T, is unclear.

Objective: To compare the effects of three implementation support models on the reach (primary outcome), dose delivered, fidelity, sustainability, impact, and cost of RT4T.

Methods and analysis: We will conduct a hybrid type III implementation-effectiveness trial involving Grade 9 and 10 (aged 14 to 16 years) students from 90 secondary schools in New South Wales (NSW), Australia. Schools will be recruited across one cohort (*n*=90 in 2023, stratified by school type, socio-economic status (SES) and location, and randomised in a 1:1:1 ratio to receive one of the following levels of implementation support: i) 'Low' (training and resources), ii) 'Moderate' (training and resources + external support), or 'High' (training and resources + external support + equipment). Training includes a teacher workshop related to RT4T program content (theory and practical sessions) and the related resources. Additional support will be provided by trained Project Officers (i.e., Project Officers from five local health districts, NSW Department of Education staff, and members of the research team). Equipment will consist of a pack of semi-portable RT equipment (i.e., weighted bars, resistance bands and inverted pull up bar stands) valued at ~AUD$1,000 per school. Study outcomes will be assessed at baseline (T0), 6-months (T1), and 18-months

For peer review only - http://bmjopen.bmj.com/site/about/guidelines.xhtml

(T2). A range of quantitative (teacher logs, observations, and teacher surveys) and qualitative (semi-structured interviews with teachers) methods will be used to assess primary and secondary outcomes. Quantitative analyses will utilise logistic mixed models for dichotomous outcomes, and ordinal or linear mixed effects regression models for continuous outcomes, with alpha levels set at $p<.025$ for the outcomes and cost comparisons of the Moderate and High support arms against Low support.

Ethics and dissemination: Ethics approval has been obtained from the University of Newcastle (H-2021-0418), the NSW Department of Education (SERAP:2022215), Hunter New England Human Research Ethics Committee (2023/ETH00052), and the Catholic Schools Office. The trial has been prospectively registered at the Australian Clinical Trials registration (ACTRN12622000861752). The design, conduct and reporting will adhere to the Consolidated Standards of Reporting Trials (CONSORT) statement, the Standards for Reporting Implementation Studies (StaRI) statement, and the Template for Intervention Description and Replication (TIDieR) checklist.

Strengths and limitations of this study

Strengths of this study include:

- The number of schools involved enabling the study to ascertain feasibility in varied school contexts.
- Partnerships with local population health practitioners to work with schools as they have a lot of experience in working with schools.
- The ability to leverage relationships between schools and health promotion officers for recruitment.
- The health benefits of RT are well known and RT4T fills a gap to address how and whether health promoting programs can be integrated into school curricula and effectively scaled-up.

Limitations of the study include:

For peer review only - http://bmjopen.bmj.com/site/about/guidelines.xhtml

- Teachers will assess students' muscular fitness using the RT4T app with minimal training.
- Our expanded delivery (to involve more teachers within any school) is reliant upon 'train-the-trainer' model.
- There is potential for secondary teachers to deliver RT4T in ways that are not consistent with the original design (lack of fidelity to the intervention).
- Our study does not include a control. However, the Low support group will serve as RT4T 'usual practice'.
- Only student-level data in a sub-set of schools is collected and given the proposed 'train-the-trainer' model, secondary teachers who collect student level data may introduce measurement bias and compromise internal validity.
- As we adapt RT4T for scale up, increasing its reach and implementing different models of support, we are fully aware of the likelihood of voltage drop (diminished impact at the student level).
- The Moderate and High implementation support groups are quite similar in what they include.

For peer review only - http://bmjopen.bmj.com/site/about/guidelines.xhtml

INTRODUCTION

Physical inactivity has been described as a global health issue and is the fourth leading cause of premature death worldwide[3]. The World Health Organisation (WHO) recommends children and adolescents participate in 60 minutes of (predominantly aerobic) moderate-to-vigorous physical activity (MVPA) daily, as well as muscle strengthening activity at least three days per week.[4] Despite these recommendations, physical activity declines substantially throughout adolescence resulting in many adolescents failing to meet daily physical activity guidelines.[5] Fewer than 1 in 50 Australian adolescents (15 to 17 years old) meet MVPA and muscle strengthening activity recommendations.[6] Poor rates of muscle strengthening activity may explain the secular decline in muscular fitness (i.e., strength, power, and endurance) of Australian youth over the past 30 years[7] with a similar decline seen internationally.[8,9] This suggests that youth participation in muscle strengthening activity may have declined globally. Muscular fitness is associated with various markers of health and well-being among children and adolescents in both cross-sectional[10] and prospective[11] studies. This prompted our team to investigate the key role of muscle strengthening activity during the school years.

Resistance training (RT) is a specialised form of muscle strengthening activity. It can be performed with or without equipment using a variety of resistive loads, and in a range of settings (e.g., at home, school, local park, or gym/fitness centre).[12] When performed routinely, RT may lead to muscle hypertrophy (i.e., increase in muscle size), improved muscular fitness (i.e., muscle strength, power, and/or endurance), body composition (i.e., increases in fat free mass and reductions in fat mass), and mental health (i.e., self-esteem) in school-aged youth.[13] RT is also recommended within global physical activity guidelines for adults[3], as it benefits a wide range of physical and mental health outcomes (including risk of chronic disease and depression/anxiety). However, the performance of RT is often perceived as more complex than aerobic activities (e.g., walking and jogging). This may explain why

For peer review only - http://bmjopen.bmj.com/site/about/guidelines.xhtml

For peer review only

participation in RT is much lower than for MVPA during adulthood.[4] Gaining the knowledge, skills, and confidence to participate in RT sets children and adolescents up for participation in RT across the lifespan.[14] However, for decades a number of myths and misconceptions about the safety and appropriateness of strength training, have prevented it from being offered to most school-aged youth.[15] Furthermore, among adults there are a number of reported barriers to participation in RT, including lack of confidence,[16] low self-efficacy,[16] lack of time,[17] and perceived lack of access to necessary equipment.[17] These barriers may also be present for adolescents, as they likely lack the knowledge, skills and/or confidence to engage in RT despite their desire to try a broader range of physical activities.[18] It is therefore necessary that adolescents are provided with ample opportunities to develop the skills, knowledge and confidence to engage in RT.

Schools are effective settings for health promotion as they provide access to most children and adolescents, and have the facilities, equipment and qualified staff needed to deliver interventions.[19,20,21] However, few school-based interventions provided adolescents with the confidence and competence to participate safely in RT[22] and no prior studies have done so at scale.[22,23] 'Scale up' is defined as the 'deliberate effort to increase the impact of successfully tested health interventions to benefit more people'.[24] Few physical activity interventions have progressed beyond efficacy testing and no studies investigating scaled up RT interventions among secondary students appear within the extant literature.[25] The WHO has advised policy makers, funders, and researchers to focus their efforts on scaling up evidence-based physical activity programs whilst exploring various models of implementation.[26] Prior to investing in scale up, evaluating the effectiveness of models of implementation to inform future scale up is necessary to guide the efficient use of resources. 'Voltage drop' is associated with diminishing effectiveness when programs are implemented at a larger scale.[27,28] Evaluating different models of implementation will help to minimise this phenomenon.

For peer review only - http://bmjopen.bmj.com/site/about/guidelines.xhtml

RT4T is an evidence-based program designed to provide adolescents with foundational knowledge about RT and enhance competence, confidence, and motivation to engage in RT across the lifespan. It was also designed to align with the Australian Curriculum for Health and Physical Education to develop the movement skills and concepts that will enable students to participate in physical activities that contribute to their health and wellbeing.[29] We conducted a rigorous evaluation of the RT4T intervention using a cluster randomised controlled trial (RCT) in 16 schools (N = 607) between 2015-2016.[1] The intervention resulted in immediate and sustained improvements in upper body muscular fitness and RT skill competency, demonstrating an effective and scalable approach to delivering RT within secondary schools. During this period, we established a partnership with the NSW Department of Education to scale-up the program. With support from the NSW Department of Education, 468 teachers from 249 NSW Government schools were trained to deliver the program.[1] We estimate that the program reached ~10,000 students between August 2015 and October 2020. However, interviews conducted with a sample of teachers (N =19) who delivered RT4T, identified considerable variability in program delivery. Also, several barriers to implementation emerged that included lack of support, motivation and time[30] impacting on program fidelity and potential sustainability.

Implementation support typically used for school physical activity programs, is often criticised as being a ‘train and forget’ model. It comprises initial training workshops followed by limited ongoing support for schools. This approach is unlikely to address the primary impediments to program implementation but may explain some shortcomings in whether evidence-based health promotion programs are adopted, implemented, or maintained within schools. The most effective approach that optimises implementation fidelity and may sustain efficacious programs over the longer-term remains unclear.[31] Therefore, the aim of this

For peer review only - http://bmjopen.bmj.com/site/about/guidelines.xhtml

present trial is to evaluate the effects of three implementation support models on the reach, effectiveness, dose delivered, fidelity, adoption, sustainability, impact, and cost of RT4T.

METHODS

Experimental design

We will conduct a hybrid type III implementation-effectiveness trial design[32] to evaluate the implementation and effectiveness of the RT4T intervention. We will recruit 90 secondary schools and randomise them to one of three groups: (i) Low, (ii) Moderate, or (iii) High implementation support. The Low support group will act as a control group (usual practice) given it is – the professional development model traditionally used by the NSW Department of Education and the delivery model used for RT4T from 2015-2020. Implementation outcomes will be conducted at baseline (T0), 6-months post baseline (T1) and 18-months post baseline (T2). Our implementation trial is guided by expert recommendations[33] and is registered with the Australian New Zealand Clinical Trials Registry (ACTRN12622000861752). The design, conduct and reporting will adhere to the Consolidated Standards of Reporting Trials (CONSORT)[34] statement, the Standards for Reporting Implementation Studies (StaRI)[35] statement, and the Template for Intervention Description and Replication (TIDieR) checklist.[36]

School recruitment and selection

Secondary schools (Government, Catholic and Independent) in ten local health districts in NSW (Hunter New England, Illawarra Shoalhaven, Nepean Blue Mountains, Northern NSW, Northern Sydney, South Western Sydney, Sydney South Eastern, Sydney, Western NSW and Western Sydney) will be eligible to participate in this study. Schools outside these regions will be able to attend the professional learning workshops and implement RT4T, but will not be recruited into the study to assess implementation outcomes. Eligible schools will be those

For peer review only - http://bmjopen.bmj.com/site/about/guidelines.xhtml

that include students in Grades 9 and 10 (aged 14 to 16 years). We will use a range of evidence-based recruitment and retention strategies to maximise participation and minimise drop-out (at both school and teacher levels), including promotion within the NSW Department of Education School Sport Unit newsletter. This will include pre-notification, the use of a dedicated recruitment coordinator, repeated reminders, and deployment of staff from local health promotion teams to engage with schools.

Participants

Teachers: One teacher from each enrolled school that teaches students in Grade 9 and 10 will be recruited to act as a 'school champion'. They will attend a single full-day professional learning workshop delivered by members of the research team with tertiary qualifications in PE, health promotion, exercise physiology and/or strength and conditioning. School champions will receive curriculum materials and resources at the workshop and will be asked to deliver a separate 2-hour training for other Grade 9 and 10 teachers at their schools, who also have the option to implement the program in their classes. Teachers will be trained to deliver the RT4T sessions to their students during usual PE lessons, co-curricular school sport periods or within an elective subject known as Physical Activity and Sports Studies. Professional learning will align with the NSW Educational Standards Authority accreditation process to incentivise teachers to participate. Lastly, the learning will contribute to teachers' required annual training hours on a yearly basis.[37,38]

Students: Secondary students in Grades 9 and 10 (aged 14 to 16 years) enrolled in the study schools will be eligible to participate.

Primary and secondary outcomes

Teachers blinded to group allocation will utilise a range of quantitative and qualitative methods to assess primary and secondary outcomes at baseline (T0), 6-months (T1) and 18-

For peer review only - http://bmjopen.bmj.com/site/about/guidelines.xhtml

months. Reach (the proportion of the eligible student population to participate in the program) is the primary implementation outcome. Secondary outcome measures include, dose delivered, fidelity, sustainability, impact, cost effectiveness, and implementation determinants.

Sample size calculation

We aim to recruit 90 secondary schools, with 30 schools assigned to each of the three treatment arms. This will include an estimated 261 classes and *N*=~7,800 students across the three arms of the trial. Based on our previous research in secondary schools, we estimate a 70% response rate from secondary school teachers after initial contact and 30% attrition rate at follow up.[39] Assuming a conservative intraclass correlation coefficient (ICC) estimate of 0.2, and a reach of 12.5% in the Low support arm, sample size will be sufficient to detect an absolute increase of 12.5% in program reach between the Low support arm and the Moderate and High support arms, with 90% power and a type I error rate of .025. For the continuous secondary student impact outcomes, power calculation was a best guess estimate. This will give the study 80% power to detect significant small-to-moderate effects (Cohen's $d = 0.4$) between treatment arms.

For peer review only

Blinding and randomisation

After teachers have completed the RT4T workshop, schools will be randomised to one of three implementation support arms. Schools will be stratified by school type and location and randomised using a random number producing algorithm by an independent statistical analysis service. Data analysis will be conducted by individuals blinded to group allocation.

Evidence-based intervention

RT4T is an 8-week multi-component physical activity program, including practical and theory-based lessons designed to develop adolescents' knowledge, competence, confidence,

For peer review only - http://bmjopen.bmj.com/site/about/guidelines.xhtml

and motivation to participate in muscle strengthening activity. The design and delivery of RT4T is guided by the Supportive, Active, Autonomous, Fair and Enjoyable (SAAFE) principles for organised physical activity sessions for school-aged youth.[40] The training sessions and progressions were revised based on the Youth Physical Development Centre Basic Resistance Training Curriculum developed by Radnor and colleagues.[41]

The RT4T program includes three major components: (i) school-based RT sessions (delivered by teachers), (ii) theoretical classroom sessions (delivered by teachers); and (iii) a smartphone phone and tablet app (used by students/ teachers).

School-based RT sessions

Teachers will deliver the RT4T program during their usual PE lessons, co-curricular or Physical Activity and Sports Studies. The RT4T practical sessions include four sub-components. Sub-components include: (i) movement based dynamic warm-up (5 minutes), (ii) RT skill development (GymFit: 15-20 minutes) focused on five RT movement categories (i.e., upper body push, upper body pull, lower body bilateral, lower body spilt, and core stability), (iii) choice of three different types of muscle strengthening activities: a fitness workout of the day (WOD), modified game with fitness infusion (GameFit), or a fun fitness challenge done to music (FunFit) (15-20 minutes); and (iv) cool down and static stretching (StretchFit: 5 minutes). Practical sessions will be supported using a variety of resources, including exercise cards (Figure 1) and the RT4T smartphone and tablet app (Figure 2). Teachers will be also provided with a handbook that includes all the practical activities and theoretical content.

Figure 1. RT4T Exercise cards (front and back side)

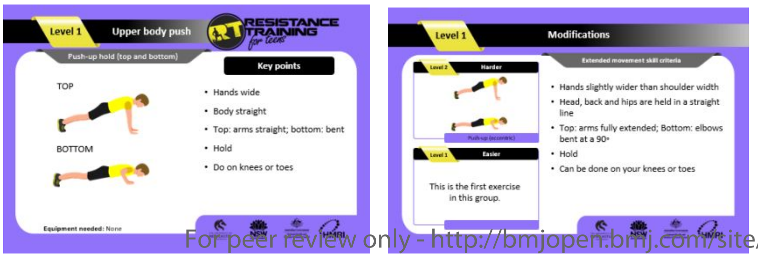

For peer review only

For peer review only - http://bmjopen.bmj.com/site/about/guidelines.xhtml

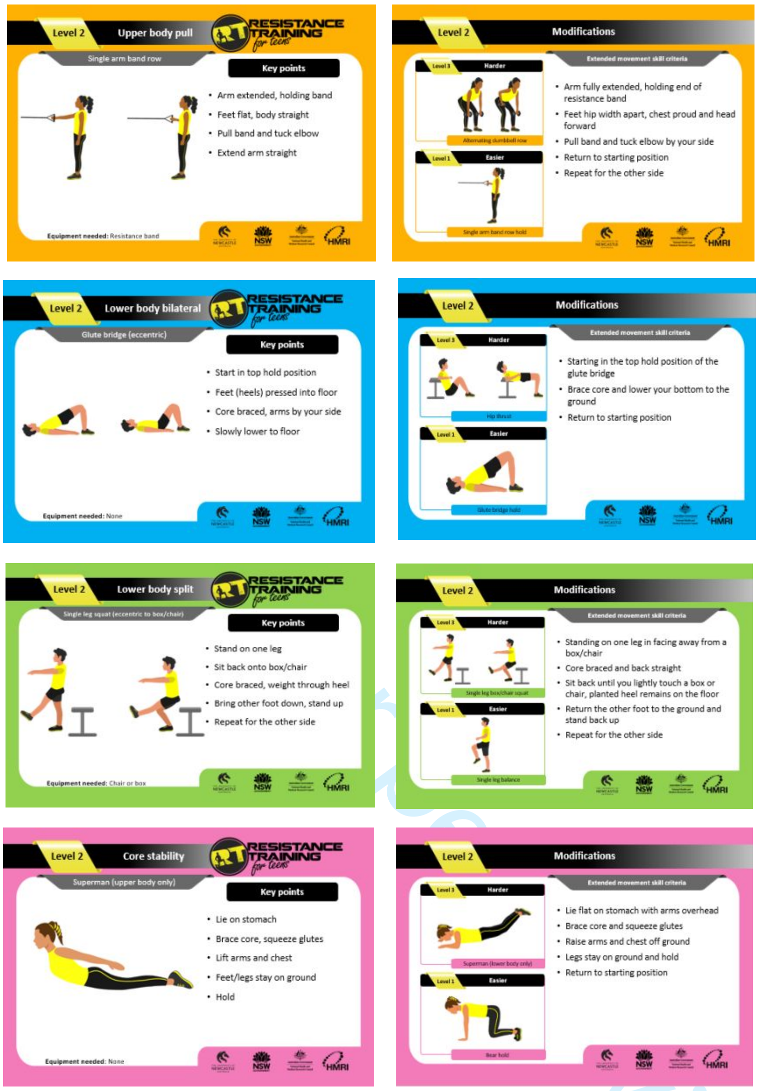

Figure 2. Resistance Training for Teens app

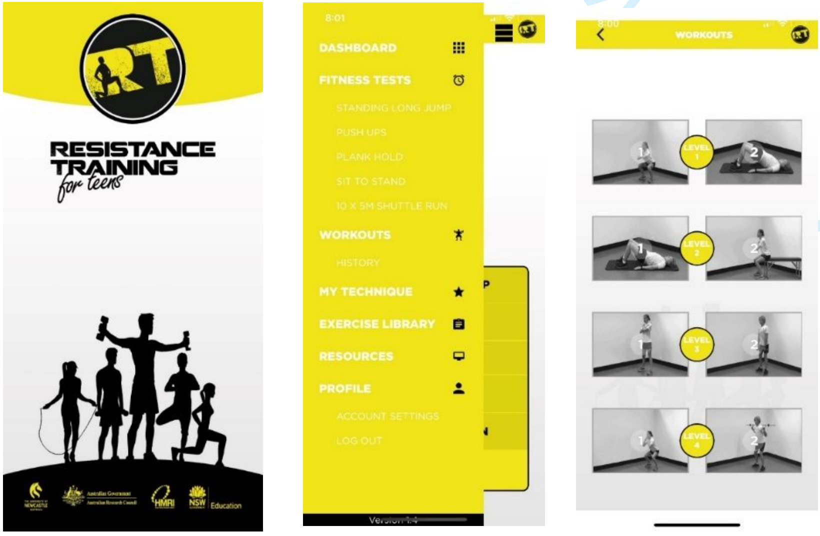

For peer review only - http://bmjopen.bmj.com/site/about/guidelines.xhtml

Theoretical classroom-based lessons

Students will receive 8 x 40-minute theory-based lessons that accompany weekly practical lessons. Content is purposefully aligned with the Australian curriculum for Health and Physical Education (HPE)[42] and Australian Physical Literacy Framework.[43] The lesson topics are generally relevant to RT and adolescent health. Topics include: (i) introduction to RT, (ii) fitness, self-assessment, and self-monitoring, (iii) fitness and e-Health technologies, (iv) principles of training and RT program design, (v) nutrition and RT: myths and recommendations, (vi) social media, physical activity, and body image, (vii) yoga, Pilates and other community-based muscle strengthening activity; and (vii) reviewing fitness outcomes and realigning future goals.

Smartphone app

We have developed and updated IOS versions of the RT4T smartphone and tablet app to support teachers to implement the program (see Figure 1). Use of the app is optional and can be downloaded from the Apple app store on all IOS devices and will be utilised for the purpose of tracking fitness test data, providing options for workouts with visual demonstrations for exercise technique along with further resources. The app includes: (i) fitness testing and criterion-referenced standards, (ii) skill testing for evaluating RT skill competence, (iii) workouts pre-designed for random selection of bodyweight RT exercises organised by difficulty using a custom workout generator; and (iv) an exercise library enabling users to browse bodyweight exercises organised by the five RT movement categories.

Implementation strategies that support teachers to deliver RT4T

We will assess whether implementation strategies can be implemented effectively across three study arms that offer implementation support. We define implementation strategies as

For peer review only - http://bmjopen.bmj.com/site/about/guidelines.xhtml

methods or techniques used to enhance adoption, implementation, or sustainability of the evidence-based intervention.[44] Study arms are (i) Low support or 'usual practice' (training and resources), (ii) Moderate support (training and resources + external support); and (iii) high support (training and resources + external support + equipment) (Table 1). Implementation strategies were co-developed with our partners in the NSW Department of Education and NSW local health districts and guided by findings from our previous dissemination study,[30.] Our strategies align with the taxonomy of strategies as described by Proctor et. al.[44]

Implementation framework and strategies

Our approach is guided by the Consolidated Framework for Implementation Research which describes constructs across five domains: (i) intervention characteristics (RT4T), (ii) outer settings (NSW Department of Education), (iii) inner setting (government schools); and (iv) individual (student/teacher) characteristics, and (v) the process of implementation.[45] We describe relevant implementation strategies in Table 1. We categorised implementation strategies as per the Consolidated Framework for Implementation Research constructs and specified their usage using the Action, Actor, Context, Target, Time framework.[46]

For peer review only - http://bmjopen.bmj.com/site/about/guidelines.xhtml

Table 1: Domains, constructs, and strategies used in our three implementation support models

Domains	Constructs	Strategies	Level of support		
			Low	Moderate	High
RT4T intervention	Evidence strength/quality	Findings from the RT4T cluster RCT were used in promotional and training materials.	✓	✓	✓
	Adaptability	Flexible intervention delivery model (PE, school sport or elective) requiring minimal access to equipment.	✓	✓	✓
	Design quality and packaging	Program resources developed by a professional graphic designer.	✓	✓	✓
Outer setting	Partnerships and investment	Investment from, and partnership with, key stakeholders from education (i.e., NSW Department of Education) and health (i.e., NSW Health local health districts).	✓	✓	✓
	External policy and incentives	Professional learning accreditation with national and state-based educational standards authorities.<br>Alignment with state school physical activity policy.	✓	✓	✓
Inner setting	School culture	School champions will run a 2-hour face-to-face professional development workshop for other teachers in their school on RT4T. Prior to this, the teachers will have completed a 30-minute pre-learning session on resistance training safety. In the 2-hour workshop the school champion will:<br>● Present the evidence and the rationale of RT4T.<br>● Teachers will participate in RT4T activities, discuss the content, and gain consensus on how they will deliver RT4T.<br>● The school champion will share and explain the resources they received at the school champion workshop.		✓	✓
	Leadership engagement	School champions and Project Officers will meet with the school principal to ensure commitment. In this meeting they will:<br>● show the 4-minute promo video which explains the benefits of RT, evidence of RT4T and an overview of the project;<br>● discuss how RT4T will be delivered in the school, who is involved, the contact person, time commitment and the costs; and<br>● complete the teacher (from all teachers who will deliver RT4T) and principal informed consents and return to the research team by sending to: RTforTeens@newcaste.edu.au		✓	✓
	Resources	Schools will receive exercise technique and workout resources. These will be provided to the school champion at the teacher workshop. All materials will also be provided on a USB which school champions will be asked to upload on to a shared drive at schools.	✓	✓	✓

For peer review only

	Equipment	Schools provided with a basic RT equipment pack (~$1,000 AUD). This includes 3x 5kg weighted bars, 3 x 8kg weighted bars, 2 x portable pull up bar stand, and 6 x sets of resistance bands. These will be delivered to the schools in the High support group following randomisation after the workshop.			✓
**Individuals**	Competence, opportunity, motivation<br>(teacher)	Full-day professional development workshop will be provided to school champions. This workshop will be accredited with the NSW Educational Standards Authority and counts towards teachers professional learning. This will be delivered by the research team (UoN) who are experts in resistance training and school interventions. These will be held for groups of schools in the local area convenient to their location. Teacher relief for the school champions will be provided by the School Sport Unit to the school to cover the cost of their attendance. Prior to attending the workshop, school champions will complete the 30-minute online pre-learning on resistance training safety. The full-day workshop training will cover the following:<br>● Rationale for RT4T (i.e., prevalence of physical inactivity in youth, benefits of RT, and myths/facts regarding participation in RT).<br>● Adaptability of RT4T.<br>● Skills and demonstrations of the movements.<br>● RT4T lesson content and alignment with the Personal Development, Health, and Physical Education syllabus.<br>● Roles and responsibilities of school champions, teachers, and Project Officers.<br>● Introduction to the RT4T app.	✓	✓	✓
	Perceived barriers (student)	Addresses student motivation using SAAFE principles.<br>● Teachers will learn to deliver RT4T using the SAAFE principles.	✓	✓	✓
**Implementation process**	Planning for implementation	Teachers will complete an action plan template to support implementation of RT4T in their school. The action plan will include:<br>● When they are going to train their staff and how.<br>● When they are going to get resources to teachers and how.<br>● Roles and responsibilities of the school champion and teachers.<br>● School champions will complete the action plan during the training.	✓	✓	✓
	Project Officers	Schools will be allocated a Project Officer (i.e., Project Officers from the local health district, NSW Department of Education staff, or member of the research team) who will provide ongoing support to the school champions after they have attended the workshop. The Project Officer will:<br>● Attend the in person principal meeting with the school champion to gain principal commitment (only if this is not done before workshop already).<br>● Email/phone/visit the school champions.<br>● Complete the checklist developed by the research team.		✓	✓

For peer review only - http://bmjopen.bmj.com/site/about/guidelines.xhtml

		• Stay in touch with the project manager from RT4T and provide all the needed details that are requested by the research team (e.g., keep track of the support provided).			
	Evaluation and feedback	The Project Officers will conduct RT4T session observations from one or two teachers per school and provide feedback on the RT4T structure.<br>• Observations will be conducted using a standardised checklist developed by the research team that assess aspects of the RT4T session (e.g., did the teachers conduct the warm-up?).<br>• Project Officers will discuss their observation results with teachers.		✓	✓

Note. RT4T = Resistance Training for Teens; SAAFE = supportive, active, autonomous, fair, and enjoyable

For peer review only

Professional learning for teachers

The school champion will complete a professional pre-learning online course prior to program delivery. This will include modules covering foundational principles of RT and specific safety recommendations to deliver RT to school-aged youth. Following initial completion of the online learning, the school champion will attend a single full-day workshop covering the background/rationale, structure, and implementation of the RT4T program, delivered by members of the research team with relevant expertise in physical education, health promotion, exercise physiology, and strength and conditioning.

School champions will be asked to attend the professional learning workshop and deliver a compressed training module to other Grade 9 and 10 teachers within their schools. The compressed module comprises a train-the-trainer approach that is supported by resources (i.e., presentation slides) provided by the research team. Our goal is to enable all interested and eligible teachers to deliver the RT4T program to maximise the reach of the RT4T program across the eligible student population.

Project Officers

Following the teacher workshop, schools randomised to the Moderate and High support groups will receive external support from Project Officers (i.e., Project Officers from five local health districts, NSW Department of Education staff, and members of the research team). Project Officers with relevant qualifications in physical education, health promotion, exercise physiology, and/or strength and conditioning will observe teachers' implementation of the RT4T practical sessions, provide ongoing support, and address the various barriers to program implementation. School champions and Project Officers in the Moderate and High support groups will meet with school leaders (principal or head of department) to ensure commitment to the RT4T program.

For peer review only - http://bmjopen.bmj.com/site/about/guidelines.xhtml

Materials and equipment

All participating schools will receive 100 hardcopy exercise cards to use during practical sessions (Figure 1). High implementation support will also receive a basic RT equipment pack. Each equipment pack will include a variety of low cost, versatile exercise equipment (e.g., rubber tubing resistance bands, exercise mats and free weights).

Measures and data collection

Quantitative assessments will be conducted at baseline (T0), 6-months (post intervention; T1) and 18-months (1 year post intervention; T2). Teachers will complete an online survey (T0, T1 and T2). Personal Development, Health, and Physical Education Heads of Department (N=18) and school champions (N=18) will be invited to participate in face-to-face or virtual interviews (T1).

Implementation evaluation

Primary implementation outcome

We define an implementation outcome as the intentional actions to deliver a policy or an intervention[47,48]. Program reach is our primary implementation indicator (outcome).

Reach: Program 'reach' is defined as the proportion and representativeness of Grade 9 and 10 students attending schools with school champions who deliver the RT4T program (≥50% of the practical sessions). We will assess reach by collecting teacher logbooks (i.e., attendance lists) at T1 and T2. We have used logbooks previously in school-based health promotion trials[49,50] and achieved high completion rates, good validity, and reliability.[51] A research team member will request that school champions provide a copy of parts of teacher logbooks that describe details of the RT4T program. Proportion will be calculated as a percentage (%) of students from Grade 9 and 10 who participate in RT4T divided by the

For peer review only - http://bmjopen.bmj.com/site/about/guidelines.xhtml

number of students in Grade 9 and 10 at selected schools. Teachers at designated schools will be asked to record students' participation in RT4T using the RT4T app.[30]

Secondary implementation outcomes

Dose delivered: Dose delivered is the number of RT4T practical and theory lessons delivered to students. Teachers will be asked to record this information in the RT4T teacher handbooks.

Fidelity*:* Project Officers will observe approximately two teachers per school deliver an RT4T practical session (total observations N = ~180). Session fidelity will be assessed by completing an observation checklist that describes the RT4T structure and process (e.g., did the teacher include a warm-up?) and to what extent they implemented the SAAFE principles (e.g., teacher was supportive and promoted positive student interactions, with answer options ranging from 1 = '*Strongly disagree'* to 4 = '*Strongly agree'*).

Sustainability*:* We define sustainability as the extent to which the program is embedded within school practices. It will be assessed in two ways. First, through an interview with the Personal Development, Health, and Physical Education, Head of Department (at T2). Second, school champions will be asked to complete the PRESS sustainability tool.[52]

Implementation determinants: We define implementation determinants as factors believed or empirically shown to influence implementation outcomes.[53] We will assess the following determinants: culture, acceptability, feasibility, adaptability, compatibility (appropriateness), dose (satisfaction), capabilities, opportunities, and motivations (COM-B) using a teacher survey (~20-minutes).

Impact evaluation

We will assess student-level outcomes in a randomly selected sample of students: fitness, participation in muscle strengthening activity, RT skill competency and muscular endurance

For peer review only - http://bmjopen.bmj.com/site/about/guidelines.xhtml

using the RT4T app. The tablet version of the app (adapted from the 'Burn 2 Learn' program)[54] will be used by teachers to assess students' fitness, student's participation in muscle strengthening activity and RT skill competency. We will evaluate upper body muscular endurance using the 90-degree push-up test[55]. Isometric abdominal muscular endurance will be measured using the plank hold test.[56] Standing long jump will assess muscular power, 1-minute sit to stand will assess lower body muscular endurance and the 10 x 5m shuttle run will assess speed and agility. Assessments are integrated into the RT4T program and conducted during the 8-week intervention period.

Statistical analysis

Statistical analyses will be conducted by an independent statistical analysis service - Clinical Research Design, Information Technology and Statistical Support from Hunter Medical Research Institute. Analyses of the primary (program reach) and secondary outcomes will be conducted using logistic mixed models (SAS Institute Inc, Cary, NC) for dichotomous outcomes, and ordinal or linear mixed effects regression models for continuous outcomes, with alpha levels set at $p<0.025$ for the comparisons of the Moderate and High support arms against the Low support arms. If the *p*-values for the differences between the Moderate and High support arms with the Low support arm reach this threshold, the Moderate and High support arms will be compared at a 5% significance threshold. For student level outcomes, statistical analyses will be adjusted for the clustering of effects at the class level, as students from each school are nested in classes. Although clustering at the school level is negligible after adjusting for clustering at the class level, we will test this assumption and adjust our analyses for school-level clustering if required. For teacher-level outcomes, clustering will be accounted for at the school-level. Three potential moderators of effects will be explored using interaction terms (i.e., type of Project Officer, socio-economic status of school, and school location).

For peer review only - http://bmjopen.bmj.com/site/about/guidelines.xhtml

Data Monitoring

All entered data will be deidentified using participant codes and will be stored electronically within the RT4T app, and in a password protected drive at the University of Newcastle. Data will be checked for implausible values, and 20% of the data will be entered twice to confirm accuracy. It is not expected that participants will be any greater risk of adverse events than they would be when participating in other types of school-based physical activity. However, the teacher handbook includes a section for teachers to report any adverse events that may occur. Any adverse events will be documented and reported to the relevant ethics committee. Any amendments to the study protocols will be publicly available via the Australian and New Zealand Clinical Trials Registry (trial number: ACTRN12622000861752).

Economic evaluation

We will conduct an economic evaluation to assess the cost effectiveness and cost-consequences of RT4T across the three trial arms. The results of the cost-consequence analysis are presented as a scorecard comprising the total incremental cost of delivering the intervention alongside the range of outcomes reflected in the primary and secondary trial outcomes (consequences). The trial-based cost-effectiveness analysis will align the cost of the intervention against the primary outcomes for each phase, age, and time point to generate an incremental cost-effectiveness ratio (ICER):

$$ICER = (⟦cost⟧ \text{ intervention} - ⟦cost⟧\_(\text{usual practice}))/(⟦effectiveness⟧ \text{ intervention} - ⟦effectiveness⟧ (\text{usual practice}))$$

Costs comprise the resource use associated with the intervention and implementation, while research costs are excluded. The opportunity cost for teacher and staff time will be prospectively measured and valued using pro-rata Department of Education salary levels. The ICER will be calculated for each comparison to determine the highest value strategy. A

For peer review only - http://bmjopen.bmj.com/site/about/guidelines.xhtml

probability sensitivity analysis will assess the value of future research to further optimise NSW Department of Education and NSW Ministry of Health spending into the most cost-effective implementation model for scale up across Australia. The incremental cost per student will be compared against the benefit achieved at the participant level across each group.

ETHICS AND DISSEMINATION

Ethics approval has been obtained from the University of Newcastle (H-2021-0418), the NSW Department of Education (SERAP:2022215), Hunter New England Human Research Ethics Committee (2023/ETH00052), and the Catholic Schools Office. The RT4T program will be delivered to students during lesson time. Therefore, they will not be required to provide written informed consent. Students outside schools within the five indicated local health districts will need to provide opt-out consent if they do not want their fitness data collected within the RT4T app.

Contributors: HTK: software, resources, writing - initial draft, writing - review and editing. JJS: conceptualisation, methodology, software, resources, writing – initial draft, writing - review and editing, supervision and funding acquisition. AV: methodology, software, resources, writing – initial draft, writing – review and editing, supervision, project administration. SGK: conceptualisation, methodology, resources, writing – original draft, writing – review and editing, funding acquisition. JS: conceptualisation, methodology, resources, writing – original draft, writing – review and editing, funding acquisition. HM: conceptualisation, methodology, software, writing – original draft, writing – review and editing, funding acquisition. NN: conceptualisation, methodology, writing – original draft, writing – review and editing, funding acquisition. RS: conceptualisation, methodology, writing – original draft, writing – review and editing, funding acquisition. PJM:

For peer review only - http://bmjopen.bmj.com/site/about/guidelines.xhtml

conceptualisation, methodology, writing – original draft, writing – review and editing, funding acquisition. JS: conceptualisation, methodology, writing – original draft, writing – review and editing, funding acquisition. DP: conceptualisation, methodology, writing – original draft, writing – review and editing, funding acquisition. JB: conceptualisation, methodology, writing – review and editing, funding acquisition. RSL: methodology, resources, writing – review and editing. CO: methodology, writing – review and editing. PR: methodology, review and editing. KP: methodology, resources, writing – review and editing. MH: writing – review and editing, project administration. SL: writing – review and editing, project administration. JN: writing – review and editing, project administration. AV: writing – review and editing, project administration. AZ: writing – review and editing, project administration. DRL: conceptualisation, methodology, software, resources, writing – original draft, writing – review and editing, supervision and funding acquisition.

Funding: This project is funded by a National Health and Medical Research Council (NHMRC) Partnership Grant (APP2010866). DRL is funded an NHMRC Senior Research Fellowship (APP1154507). NN is funded by the Medical Research Future Fund (MRFF) Investigator Grant (GS2000053). This project is supported and co-designed with the NSW Department of Education.

Disclaimer: The study funders will have no role in data collection, analysis, interpretation, or writing nor will they have influence over the publication of findings.

Competing interests: None declared.

Patient and public involvement: The NSW Department of Health, NSW local health districts, secondary school teachers, and students were involved in the design of the RT4T intervention.

Patient consent for publication: Not applicable.

For peer review only - http://bmjopen.bmj.com/site/about/guidelines.xhtml

Provenance and peer review: Not commissioned; externally peer reviewed.

Supplemental material: This content has been supplied by the author(s). It has not been vetted by BMJ Publishing Group Limited (BMJ) and may not have been peer-reviewed. Any opinions or recommendations discussed are solely those of the author(s) and are not endorsed by BMJ. BMJ disclaims all liability and responsibility arising from any reliance placed on the content. Where the content includes any translated material, BMJ does not warrant the accuracy and reliability of the translations (including but not limited to local regulations, clinical guidelines, terminology, drug names and drug dosages), and is not responsible for any error and/or omissions arising from translation and adaptation or otherwise.

Open access: This is an open access article distributed in accordance with the Creative Commons Attribution 4.0 Unported (CC BY 4.0) license, which permits others to copy, redistribute, remix, transform and build upon this work for any purpose, provided the original work is properly cited, a link to the licence is given, and indication of whether changes were made. See: https://creativecommons.org/ licenses/by/4.0/

ORCID IDs:

Hayden T Kelly http://orcid.org/0000-0002-4533-6945

Angeliek Verdonschot https://orcid.org/0000-0002-1519-3402

Sarah Kennedy https://orcid.org/0000-0001-9804-616X

Nicole Nathan https://orcid.org/0000-0002-7726-1714

Philip Morgan https://orcid.org/0000-0002-5632-8529

Jo Salmon https://orcid.org/0000-0002-4734-6354

Dawn Penney https://orcid.org/0000-0002-2000-8953

For peer review only

James Boyer https://orcid.org/0000-0002-0585-4238

Rhodri Lloyd https://orcid.org/0000-0001-8560-1566

Jennifer Norman https://orcid.org/0000-0001-6344-2605

Alexander Voukelatos https://orcid.org/0000-0003-3131-761X

Avigdor Zask https://orcid.org/0000-0002-3888-7335

David R Lubans http://orcid.org/0000-0002-0204-8257

References

1. Kennedy SG, Smith JJ, Morgan PJ, *et al*. Implementing resistance training in secondary schools: A cluster randomized controlled trial. ***Med Sci Sports Exerc*** 2018;50:62-72.
2. Vanhelst J, Beghin L, Fardy PS, *et al*. Reliability of health-related physical fitness tests in adolescents: the MOVE Program. ***Clin Physiol Funct Imaging*** 2016;36:106-111.
3. Kohl HW, Craig CL, Lambert EV, *et al*. The pandemic of physical inactivity: global action for public health. ***Lancet*** 2012;380:294–305.
4. Bull FC, Al-Ansari SS, Biddle S, *et al.* World Health Organization 2020 guidelines on physical activity and sedentary behaviour. ***Br J Sports Med*** 2020;54:24:1451-1462.
5. Farooq MA, Parkinson KN, Adamson AJ, *et al*. Timing of the decline in physical activity in childhood and adolescence: Gateshead Millennium Cohort Study. ***Br J Sports Med*** 2018;52:15:1002-1006.
6. Australian Bureau of Statistics. National Health Survey: First Results, 2017-18. Australian Bureau of Statistics, 2019.
7. Fraser BJ, Blizzard L, Tomkinson GR, *et al.* The great leap backward: changes in the jumping performance of Australian children aged 11−12-years between 1985 and 2015. ***J Sports Sci*** 2019;37:748–754.
8. Tomkinson GR, Kaster T, Dooley FL, *et al*. Temporal Trends in the Standing Broad Jump Performance of 10,940,801 Children and Adolescents Between 1960 and 2017. ***Sports Med*** 2021;51:531–548.
9. Ortega FB, Leskošek B, Blagus R, *et al.* European fitness landscape for children and adolescents: updated reference values, fitness maps, and country rankings based on nearly 8 million test results from 34 countries gathered by the FitBack network. ***Br J Sports Med*** 2023;57:5:299-310.
10. Smith JJ, Eather N, Morgan P.J, *et al.* The health benefits of muscular fitness for children and adolescents: a systematic review and meta-analysis. ***Sports Med*** 2014;44:1209–1223.
11. García-Hermoso A, Ramírez-Campillo R, Izquierdo M. Is Muscular Fitness Associated with Future Health Benefits in Children and Adolescents? A Systematic Review and Meta-Analysis of Longitudinal Studies. ***Sports Med*** 2019;49:1079–1094.

For peer review only - http://bmjopen.bmj.com/site/about/guidelines.xhtml

12. Lloyd RS, Faigenbaum AD, Stone MH, *et al.* Position statement on youth resistance training: the 2014 International Consensus. ***Br J Sports Med*** 2014;48:7:498–505.
13. Brusseau T, Fairclough S, Lubans DR. The Routledge Handbook of Youth Physical Activity. Oxon, UK: Routledge; 2020:738-761.
14. Faigenbaum AD, McFarland JE. Resistance training for kids: Right from the Start. ***ACSM's Health & Fit J*** 2016;20:5:16-22.
15. Faigenbaum AD, Stracciolini A, MacDonald JP, *et al.* Methodology of youth resistance training. ***Br J Sports Med*** 2022;105804.
16. Rhodes, RE, Lubans DR, Karunamuni N, *et al.* Factors associated with participation in resistance training: a systematic review. ***Br J Sports Med*** 2017;51:20:1466-1472.
17. Steele J, Fisher J, Skivington M, *et al.* A higher effort-based paradigm in physical activity and exercise for public health: making the case for a greater emphasis on resistance training. ***BMC Public Health*** 2017;17:300.
18. Tannehill D, MacPhail A, Walsh J, *et al.* What young people say about physical activity: the Children's Sport Participation and Physical Activity (CSPPA) study. ***Sport Educ Soc*** 2015;20:442-462.
19. Hills AP, Dengel DR, Lubans DR. Supporting public health priorities: recommendations for physical education and physical activity promotion in schools. ***Prog Cardiovasc Dis*** 2015;57:4:368–374.
20. Poland B, Krupa G, McCall D. Settings for health promotion: an analytic framework to guide intervention design and implementation. ***Health Promot Pract*** 2009;10:4:505-516.
21. Neufeld J, Kettner J. The Settings Approach in Public Health: Thinking about Schools in Infectious Disease Prevention and Control. ***Purple Paper*** 2014;45.
22. Cox A, Fairclough SJ, Kosteli MC, *et al.* Efficacy of School-Based Interventions for Improving Muscular Fitness Outcomes in Adolescent Boys: A Systematic Review and Meta-analysis. ***Sports Med*** 2020;50:543–560.
23. Malina RM. Weight training in youth-growth, maturation, and safety: an evidence-based review. ***Clin J Sport Med*** 2006;16:6:478-487.
24. Milat AJ, Newson R, King L, *et al.* A guide to scaling up population health interventions. ***Public Health Res Pract*** 2016;26:1:e2611604.
25. Reis RS, Salvo D, Ogilvie, D, *et al.* Scaling up physical activity interventions worldwide: stepping up to larger and smarter approaches to get people moving. ***Lancet*** 2016;388:10051:1337–1348.
26. World Health Organization. Nine steps for developing a scaling-up strategy. Geneva: World Health Organisation, 2010.
27. Chambers DA, Glasgow RE, Stange KC. The dynamic sustainability framework: addressing the paradox of sustainment amid ongoing change. ***Implement Sci*** 2013;8:117.
28. McCrabb S, Lane C, Hall A, *et al.* Scaling-up evidence-based obesity interventions: A systematic review assessing intervention adaptations and effectiveness and quantifying the scale-up penalty. ***Obes Rev*** 2019;20:7:964–982.
29. Rationale. The Australian Curriculum (Version 8.4). https://www.australiancurriculum.edu.au/f-10-curriculum/health-and-physical-education/rationale/.
30. Kennedy SG, Smith JJ, Hansen V, *et al.* Implementing Resistance Training in Secondary Schools: An Exploration of Teachers' Perceptions. ***TJACSM*** 2018;3:12:85-96.

For peer review only - http://bmjopen.bmj.com/site/about/guidelines.xhtml

31. Shoesmith A, Hall A, Wolfenden L, *et al.* Barriers and facilitators influencing the sustainment of health behaviour interventions in schools and childcare services: a systematic review. ***Implement Sci*** 2021;16:1:62.
32. Landes SJ, McBain SA, Curran GM. An introduction to effectiveness-implementation hybrid designs. ***Psychiatry Res*** 2019;280:112513.
33. Mckay H, Naylor P-J, Lau E, *et al.* Implementation and scale-up of physical activity and behavioural nutrition interventions: an evaluation roadmap. ***Int J of Behav Nutr Phys Act*** 2019;16:1:102.
34. Department of Health. Australia's Long Term National Health Plan. Canberra: Australian Government, 2019.
35. Pinnock H, Barwick M, Carpenter CR, *et al.* Standards for Reporting Implementation Studies (StaRI) Statement. ***BMJ*** 2017;356:i6795.
36. Wolfenden L, Kypri K, Freund M, *et al.* Obtaining active parental consent for school-based research: a guide for researchers. ***Aust N Z J Public Health*** 2009;33:3:270-275.
37. Kennedy SG, Sanders T, Estabrooks PA, *et al.* Implementation at-scale of school-based physical activity interventions: A systematic review utilizing the RE-AIM framework. ***Obes Rev*** 2021;22:7:e13184.
38. Sutherland RL, Campbell EM, Lubans DR, *et al.* The Physical Activity 4 Everyone Cluster randomized Trial: 2-Year Outcomes of a School Physical Activity Intervention Among Adolescents. ***Am J Prev Med*** 2016;51:2:195-205.
39. Kennedy SG, Peralta LR, Lubans DR, *et al.* Implementing a school-based physical activity program: process evaluation and impact on teachers' confidence, perceived barriers and self-perceptions. ***Phys Educ Sport Pedagogy*** 2019;24:3:233-248.
40. Lubans DR, Lonsdale C, Cohen K, *et al.* Framework for the design and delivery of organized physical activity sessions for children and adolescents: rationale and description of the 'SAAFE' teaching principles. ***Int J Behav Nutr Phys Act*** 2017;14:1:24.
41. Radnor JM, Moeskops S, Morris SJ, *et al.* Developing Athletic Motor Skill Competencies in Youth. ***Strength Cond J*** 2020;42:6:54-70.
42. Australian Curriculum, Assessment and Reporting Authority. ACARA - Curriculum, 2022. Retrieved March 27, 2023, from https://acara.edu.au/curriculum.
43. Keegan R, Barnett L, Dudley D. The Physical Literacy Framework. Sport Australia, 2019. Retrieved March 27, 2023, from https://www.sportaus.gov.au/__data/assets/pdf_file/0019/710173/35455_Physical-Literacy-Framework_access.pdf.
44. Proctor EK, Powell BJ, McMillen JC. Implementation strategies: recommendations for specifying and reporting. ***Implement Sci*** 2013;8:139.
45. Keith RE, Crosson JC, O'Malley AS, *et al.* Using the Consolidated Framework for Implementation Research (CFIR) to produce actionable findings: a rapid-cycle evaluation approach to improving implementation. ***Implement Sci*** 2017;12:1:15.
46. Presseau J, McCleary N, Lorencatto F, *et al.* Action, actor, context, target, time (AACTT): a framework for specifying behaviour. ***Implement Sci*** 2019;14:1:102.
47. Proctor EK, Silmere H, Raghavan R, *et al.* Outcomes for implementation research: conceptual distinctions, measurement challenges, and research agenda. ***Adm Policy Ment Health*** 2011;38:2:65-76.
48. Peters DH, Adam T, Alonge O, *et al*. Implementation research: what it is and how to do it. ***BMJ*** 2014;48:8:731-736.

For peer review only - http://bmjopen.bmj.com/site/about/guidelines.xhtml

49. Nathan NK, Hall A, McCarthy N, *et al.* Multi-strategy intervention increases school implementation and maintenance of a mandatory physical activity policy: outcomes of a cluster randomised controlled trial. ***Br J Sports Med*** 2022;56:7:385-393.
50. Nathan NK, Sutherland RL, Hope K, *et al.* Implementation of a School Physical Activity Policy Improves Student Physical Activity Levels: Outcomes of a Cluster-Randomized Controlled Trial. ***J Phys Act Health*** 2020;17:10:1009-1018.
51. Cradock AL, Barrett JL, Carter J, *et al*. Impact of the Boston Active School Day Policy to promote physical activity among children. ***Am J Health Promot*** 2014;28:3:54-64.
52. Moullin JC, Sklar M, Ehrhart MG, *et al.* Provider REport of Sustainment Scale (PRESS): development and validation of a brief measure of inner context sustainment. ***Implement Sci*** 2021;16:86.
53. Nilsen P, Bernhardsson S. Context matters in implementation science: a scoping review of determinant frameworks that describe contextual determinants for implementation outcomes. ***BMC Health Serv Res*** 2019;19:1:189.
54. Lubans DR, Smith JJ, Eather N, *et al.* Time-efficient intervention to improve older adolescents' cardiorespiratory fitness: findings from the 'Burn 2 Learn' cluster randomised controlled trial. ***Br J of Sports Med*** 2021;55:751–758.
55. Lubans DR, Morgan P, Callister R, *et al.* Test–retest reliability of a battery of field-based health-related fitness measures for adolescents. ***J Sports Sci*** 2011;29:7:685–693.
56. Boyer C, Tremblay M, Saunders TJ, *et al*. Feasibility, validity and reliability of the plank isometric hold as a field-based assessment of torso muscular endurance for children 8-12 years of age. ***Paediatr Exerc Sci*** 2013;25:3:407-422.

For peer review only

For peer review only - http://bmjopen.bmj.com/site/about/guidelines.xhtml

Table 1: Domains, constructs, and strategies used in our three implementation support models

Domains	Constructs	Strategies	Level of support		
			Low	Moderate	High
**RT4T intervention**	Evidence strength/quality	Findings from the RT4T cluster RCT were used in promotional and training materials.	✓	✓	✓
	Adaptability	Flexible intervention delivery model (PE, school sport or elective) requiring minimal access to equipment.	✓	✓	✓
	Design quality and packaging	Program resources developed by a professional graphic designer.	✓	✓	✓
**Outer setting**	Partnerships and investment	Investment from, and partnership with, key stakeholders from education (i.e., NSW Department of Education) and health (i.e., NSW Health local health districts).	✓	✓	✓
	External policy and incentives	Professional learning accreditation with national and state-based educational standards authorities. Alignment with state school physical activity policy.	✓	✓	✓
**Inner setting**	School culture	School champions will run a 2-hour face-to-face professional development workshop for other teachers in their school on RT4T. Prior to this, the teachers will have completed a 30-minute pre-learning session on resistance training safety. In the 2-hour workshop the school champion will:<br>● Present the evidence and the rationale of RT4T.<br>● Teachers will participate in RT4T activities, discuss the content, and gain consensus on how they will deliver RT4T.<br>● The school champion will share and explain the resources they received at the school champion workshop.		✓	✓
	Leadership engagement	School champions and external change agents will meet with the school principal to ensure commitment. In this meeting they will:<br>● show the 4-minute promo video which explains the benefits of RT, evidence of RT4T and an overview of the project;<br>● discuss how RT4T will be delivered in the school, who is involved, the contact person, time commitment and the costs; and<br>● complete the teacher (from all teachers who will deliver RT4T) and principal informed consents and return to the research team by sending to: RTforTeens@newcaste.edu.au		✓	✓
	Resources	Schools will receive exercise technique and workout resources. These will be provided to the school champion at the teacher workshop. All materials will also be provided on a USB which school champions will be asked to upload on to a shared drive at schools.	✓	✓	✓

For peer review only - http://bmjopen.bmj.com/site/about/guidelines.xhtml

	Equipment	Schools provided with a basic RT equipment pack (~$1,000 AUD). This includes 3x 5kg weighted bars, 3 x 8kg weighted bars, 2 x portable pull up bar stand, and 6 x sets of resistance bands. These will be delivered to the schools in the High support group following randomisation after the workshop.			✓
**Individuals**	Competence, opportunity, motivation<br>(teacher)	Full-day professional development workshop will be provided to school champions. This workshop will be accredited with the NSW Educational Standards Authority and counts towards teachers professional learning. This will be delivered by the research team (UoN) who are experts in resistance training and school interventions. These will be held for groups of schools in the local area convenient to their location. Teacher relief for the school champions will be provided by the School Sport Unit to the school to cover the cost of their attendance. Prior to attending the workshop, school champions will complete the 30-minute online pre-learning on resistance training safety. The full-day workshop training will cover the following:<br>● Rationale for RT4T (i.e., prevalence of physical inactivity in youth, benefits of RT, and myths/facts regarding participation in RT).<br>● Adaptability of RT4T.<br>● Skills and demonstrations of the movements.<br>● RT4T lesson content and alignment with the Personal Development, Health, and Physical Education syllabus.<br>● Roles and responsibilities of school champions, teachers, and external change agents.<br>● Introduction to the RT4T app.	✓	✓	✓
	Perceived barriers (student)	Addresses student motivation using SAAFE principles.<br>● Teachers will learn to deliver RT4T using the SAAFE principles.	✓	✓	✓
**Implementation process**	Planning for implementation	Teachers will complete an action plan template to support implementation of RT4T in their school. The action plan will include:<br>● When they are going to train their staff and how.<br>● When they are going to get resources to teachers and how.<br>● Roles and responsibilities of the school champion and teachers.<br>● School champions will complete the action plan during the training.	✓	✓	✓
	External change agents	Schools will be allocated an external change agent (i.e., Project Officers from the local health district, NSW Department of Education staff, or member of the research team) who will provide ongoing support to the school champions after they have attended the workshop. The external change agents will:<br>● Attend the in person principal meeting with the school champion to gain principal commitment (only if this is not done before workshop already).<br>● Email/phone/visit the school champions.<br>● Complete the checklist developed by the research team.		✓	✓

For peer review only - http://bmjopen.bmj.com/site/about/guidelines.xhtml

		• Stay in touch with the project manager from RT4T and provide all the needed details that are requested by the research team (e.g., keep track of the support provided).			
	Evaluation and feedback	The external change agents will conduct RT4T session observations from one or two teachers per school and provide feedback on the RT4T structure.<br>• Observations will be conducted using a standardised checklist developed by the research team that assess aspects of the RT4T session (e.g., did the teachers conduct the warm-up?).<br>• External change agents will discuss their observation results with teachers.		✓	✓

Note. RT4T = Resistance Training for Teens; SAAFE = supportive, active, autonomous, fair, and enjoyable.

For peer review only

For peer review only - http://bmjopen.bmj.com/site/about/guidelines.xhtml

For peer review only

For peer review only - http://bmjopen.bmj.com/site/about/guidelines.xhtml

Figure 1. RT4T Exercise cards (front and back side)

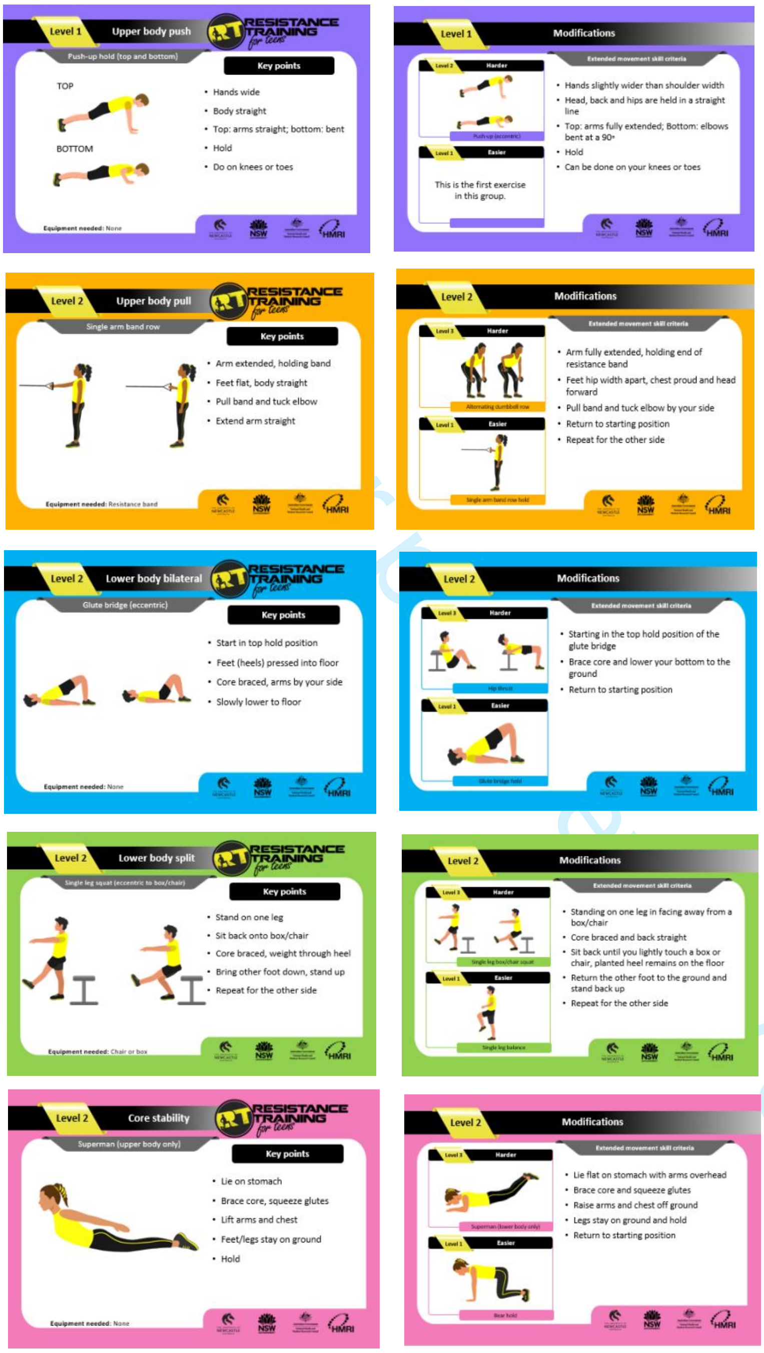

Figure 2. Resistance Training for Teens app

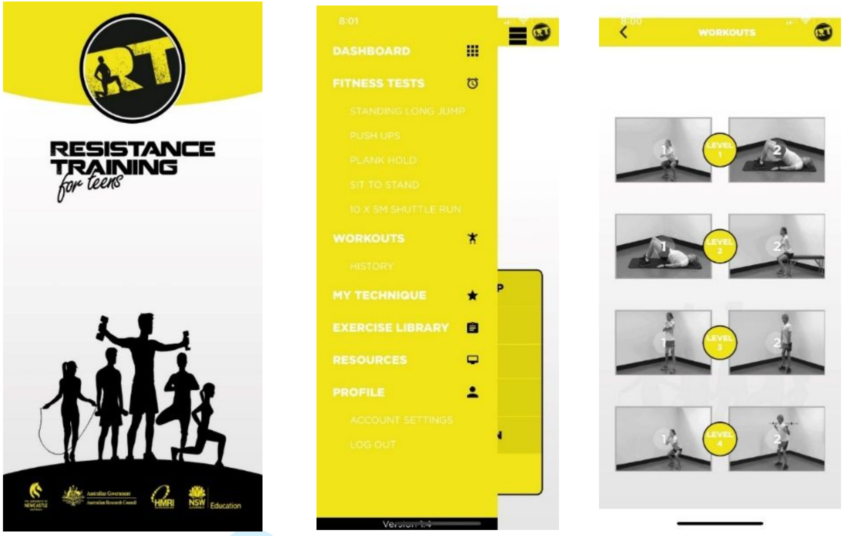

peer review only

TIDieR
Template for Intervention Description and Replication

The TIDieR (Template for Intervention Description and Replication) Checklist*:

Information to include when describing an intervention and the location of the information

Item number	Item	Where located ** Primary paper (page or appendix number)	Other † (details)
	**BRIEF NAME**		
**1.**	Provide the name or a phrase that describes the intervention.	page 2	______________
	**WHY**		
**2.**	Describe any rationale, theory, or goal of the elements essential to the intervention.	pages 7-10	______________
	**WHAT**		
**3.**	Materials: Describe any physical or informational materials used in the intervention, including those provided to participants or used in intervention delivery or in training of intervention providers. Provide information on where the materials can be accessed (e.g. online appendix, URL).	pages 10-11	______________
**4.**	Procedures: Describe each of the procedures, activities, and/or processes used in the intervention, including any enabling or support activities.	pages 10-13	______________
	**WHO PROVIDED**		
**5.**	For each category of intervention provider (e.g. psychologist, nursing assistant), describe their expertise, background and any specific training given.	pages 11,20,21	______________
	**HOW**		
**6.**	Describe the modes of delivery (e.g. face-to-face or by some other mechanism, such as internet or telephone) of the intervention and whether it was provided individually or in a group.	pages 13-14	______________
	**WHERE**		
**7.**	Describe the type(s) of location(s) where the intervention occurred, including any necessary infrastructure or relevant features.	pages 13-14	______________

For peer review only

For peer review only - http://bmjopen.bmj.com/site/about/guidelines.xhtml

	**WHEN and HOW MUCH**		
**8.**	Describe the number of times the intervention was delivered and over what period of time including the number of sessions, their schedule, and their duration, intensity or dose.	pages 13-14	______________
	**TAILORING**		
**9.**	If the intervention was planned to be personalised, titrated or adapted, then describe what, why, when, and how.	pages 13-15	______________
	**MODIFICATIONS**		
**10.‡**	If the intervention was modified during the course of the study, describe the changes (what, why, when, and how).	N/A	______________
	**HOW WELL**		
**11.**	Planned: If intervention adherence or fidelity was assessed, describe how and by whom, and if any strategies were used to maintain or improve fidelity, describe them.	pages 21-22	______________
**12.‡**	Actual: If intervention adherence or fidelity was assessed, describe the extent to which the intervention was delivered as planned.	N/A	______________

** **Authors** - use N/A if an item is not applicable for the intervention being described. **Reviewers** – use '?' if information about the element is not reported/not sufficiently reported.

† If the information is not provided in the primary paper, give details of where this information is available. This may include locations such as a published protocol or other published papers (provide citation details) or a website (provide the URL).

‡ If completing the TIDieR checklist for a protocol, these items are not relevant to the protocol and cannot be described until the study is complete.

* We strongly recommend using this checklist in conjunction with the TIDieR guide (see *BMJ* 2014;348:g1687) which contains an explanation and elaboration for each item.

* The focus of TIDieR is on reporting details of the intervention elements (and where relevant, comparison elements) of a study. Other elements and methodological features of studies are covered by other reporting statements and checklists and have not been duplicated as part of the TIDieR checklist. When a **randomised trial** is being reported, the TIDieR checklist should be used in conjunction with the CONSORT statement (see www.consort-statement.org) as an extension of **Item 5 of the CONSORT 2010 Statement.** When a **clinical trial protocol** is being reported, the TIDieR checklist should be used in conjunction with the SPIRIT statement as an extension of **Item 11 of the SPIRIT 2013 Statement** (see www.spirit-statement.org). For alternate study designs, TIDieR can be used in conjunction with the appropriate checklist for that study design (see www.equator-network.org).

For peer review only - http://bmjopen.bmj.com/site/about/guidelines.xhtml
For peer review only

BMJ Open

Supporting adolescents' participation in muscle-strengthening physical activity: Protocol for the 'Resistance Training for Teens' (RT4T) hybrid type III implementation-effectiveness trial

Journal:	*BMJ Open*
Manuscript ID	bmjopen-2023-075488.R1
Article Type:	Protocol
Date Submitted by the Author:	08-Sep-2023
Complete List of Authors:	Kelly, Hayden; The University of Newcastle, School of Education<br>Smith, Jordan ; University of Newcastle, School of Education<br>Verdonschot, Angeliek; The University of Newcastle, School of Education<br>Kennedy, Sarah; Western Sydney University, School of Health Sciences<br>Scott, Joseph; University of the Sunshine Coast; Edith Cowan University, School of Education<br>McKay, Heather; The University of British Columbia, Family Practice<br>Nathan, Nicole; Population Health Hunter New England Local Health District; Hunter Medical Research Institute<br>Sutherland, Rachel; Population Health Hunter New England Local Health District; The University of Newcastle, School of Medicine and Public Health<br>Morgan, Philip; University of Newcastle, PRC for Physical Activity and Nutrition<br>Salmon, Jo; Deakin University,<br>Penney, Dawn; Edith Cowan University, School of Education<br>Boyer, James; NSW Department of Education, School Sport Unit<br>Lloyd, Rhodri S. ; Cardiff Metropolitan University,<br>Oldmeadow, Christopher; Hunter Medical Research Institute<br>Reeves, Penny; University of Newcastle Hunter Medical Research Institute, Health Research Economics; University of Newcastle, School of Medicine and Public Health<br>Pursey, Kirrilly; The University of Newcastle, School of Health<br>Hua, Myna; Western Sydney University Translational Health Research Institute, School of Health Science<br>Longmore, Sarah; Western NSW Local Health District, Health Promotion<br>Norman , Jennifer ; University of Wollongong Illawarra Health and Medical Research Institute; Illawarra Shoalhaven Local Health District, Health Promotion Service<br>Voukelatos, Alexander; University of New South Wales, Population Health Research and Evaluation Hub, Sydney Local Health District<br>Zask, Avigdor; Northern NSW Local Health District, Health Promotion<br>Lubans, David; The University of Newcastle, School of Education
**Primary Subject Heading**:	Global health
Secondary Subject Heading:	Public health, Evidence based practice, Global health

Keywords:	Adolescent, Schools, EDUCATION & TRAINING (see Medical Education & Training)

SCHOLARONE™
Manuscripts

Supporting adolescents' participation in muscle-strengthening physical activity: Protocol for the 'Resistance Training for Teens' (RT4T) hybrid type III implementation-effectiveness trial

Hayden T, Kelly[1], Jordan J, Smith[1,2], Angeliek Verdonschot[1], Sarah G, Kennedy[3], Joseph Scott[4,5], Heather McKay[6], Nicole Nathan[2,7,8,9], Rachel Sutherland[2,7,8,9], Philip J, Morgan[1], Jo Salmon[10], Dawn Penney[11], James Boyer[12], Rhodri S, Lloyd[13], Christopher Oldmeadow[14], Penny Reeves[14], Kirrilly Pursey[15,16], Myna Hua[3], Sarah Longmore[17], Jennifer Norman[18], Alexander Voukelatos[19], Avigdor Zask[20,21], and David R, Lubans[1,2,22,*]

[1] Centre for Active Living and Learning, School of Education, University of Newcastle, Callaghan, New South Wales, Australia

[2] Hunter Medical Research Institute, New Lambton Heights, New South Wales, Australia

[3] School of Health Science, Translational Health Research Institute, Western Sydney University, Kingswood, Australia

[4] University of the Sunshine Coast, Queensland, Australia

[5] School of Education, Edith Cowan University, Perth, Western Australia, Australia

[6] University of British Columbia, Vancouver, Canada

[7] Hunter New England Population Health, Hunter New England Area Health Service, Newcastle Australia

[8] School of Medicine and Public Health, The University of Newcastle, Newcastle, New South Wales, Australia

[9] National Centre of Implementation Science, Newcastle, New South Wales, Australia

[10] Deakin University, Geelong, Victoria, Australia

[11] School of Education, Edith Cowan University, Joondalup, Australia

[12] NSW Department of Education, Sydney, New South Wales, Australia

[13] Youth Physical Development Centre, Cardiff School of Sport and Health Sciences, Cardiff Metropolitan University, Wales

[14] Hunter Medical Research Institute, College of Health, Medicine and Wellbeing, University of Newcastle, New South Wales, Australia

[15] School of Health Sciences, College of Health, Medicine and Wellbeing, University of Newcastle, Callaghan, New South Wales, Australia

For peer review only - http://bmjopen.bmj.com/site/about/guidelines.xhtml

[16] Food and Nutrition Research Group, Hunter Medical Research Institute, New Lambton Heights, New South Wales, Australia

[17] Health Promotion, Western New South Wales Local Health District, Bathurst, New South Wales, Australia

[18] Health Promotion Service, Illawarra Shoalhaven Local Health District, Warrawong, New South Wales, Australia

[19] Population Health Research and Evaluation Hub, Sydney Local Health District, School of Population Health, University of New South Wales, New South Wales Australia

[20] Health Promotion, Northern NSW Local Health District, Lismore

[21] University Centre of Rural Health (Northern Rivers), School of Public Health, University of Sydney, Lismore, NSW Australia

[22] Faculty of Sport and Health Sciences, University of Jyväskylä, Jyväskylä, Finland

Running title

RT4T implementation-effectiveness trial

***Corresponding author**

Professor David Lubans

College of Human and Social Futures, School of Education, University of Newcastle

ATC 303, Advanced Technology Centre

Callaghan, University Drive, NSW, 2308, Australia

Phone: +612 4921 2049

Email: David.Lubans@newcastle.edu.au

Word count: 5338 (excluding authorship statement, reference list and table 1)*

Keywords: School, Resistance training, Implementation, Adolescents, Physical literacy

For peer review only

ABSTRACT

Introduction: In Australia, only 22% of male and 8% of female adolescents meet the muscle-strengthening physical activity guidelines, and few school-based interventions support participation in resistance training (RT). After promising findings from our effectiveness trial, we conducted a state-wide dissemination of the '*Resistance Training for Teens'* (RT4T) intervention from 2015 to 2020. Despite high estimated reach, we found considerable variability in program delivery and teachers reported numerous barriers to implementation. Supporting schools when they first adopt evidence-based programs may strengthen program fidelity, sustainability, and by extension, program impact. However, the most effective implementation support model for RT4T, is unclear.

Objective: To compare the effects of three implementation support models on the reach (primary outcome), dose delivered, fidelity, sustainability, impact, and cost of RT4T.

Methods and analysis: We will conduct a hybrid type III implementation-effectiveness trial involving Grade 9 and 10 (aged 14 to 16 years) students from 90 secondary schools in New South Wales (NSW), Australia. Schools will be recruited across one cohort (*n*=90 in 2023, stratified by school type, socio-economic status (SES) and location, and randomised in a 1:1:1 ratio to receive one of the following levels of implementation support: i) 'Low' (training and resources), ii) 'Moderate' (training and resources + external support), or 'High' (training and resources + external support + equipment). Training includes a teacher workshop related to RT4T program content (theory and practical sessions) and the related resources. Additional support will be provided by trained Project Officers (i.e., Project Officers from five local health districts). Equipment will consist of a pack of semi-portable RT equipment (i.e., weighted bars, dumbbells, resistance bands and inverted pull up bar stands) valued at ~AUD$1,000 per school. Study outcomes will be assessed at baseline (T0), 6-months (T1), and 18-months (T2). A range of quantitative (teacher logs, observations, and

For peer review only - http://bmjopen.bmj.com/site/about/guidelines.xhtml

teacher surveys) and qualitative (semi-structured interviews with teachers) methods will be used to assess primary (reach) and secondary outcomes (dose delivered, fidelity, sustainability, impact, and cost of RT4T). Quantitative analyses will utilise logistic mixed models for dichotomous outcomes, and ordinal or linear mixed effects regression models for continuous outcomes, with alpha levels set at $p<.025$ for the outcomes and cost comparisons of the Moderate and High support arms against Low support.

Ethics and dissemination: Ethics approval has been obtained from the University of Newcastle (H-2021-0418), the NSW Department of Education (SERAP:2022215), Hunter New England Human Research Ethics Committee (2023/ETH00052), and the Catholic Schools Office. The trial has been prospectively registered at the Australian Clinical Trials registration (ACTRN12622000861752). The design, conduct and reporting will adhere to the Consolidated Standards of Reporting Trials (CONSORT) statement, the Standards for Reporting Implementation Studies (StaRI) statement, and the Template for Intervention Description and Replication (TIDieR) checklist. Findings will be published in open access peer-reviewed journals, key stakeholders will be provided with a detailed report. We will support on-going dissemination of RT4T in Australian schools via professional learning for teachers.

For peer review only

Strengths and limitations of this study

Strengths and limitation of this study include:

- Our study will test three implementation support models on a range of implementation outcomes and determinants using a hybrid type III implementation-effectiveness trial.
- Local health district staff (i.e., Project Officers) are well placed to support the implementation of school-based interventions, but few studies have examined their capacity to support secondary schools.
- There is potential for secondary school teachers to deliver RT4T with poor fidelity.

For peer review only - http://bmjopen.bmj.com/site/about/guidelines.xhtml

- Our study does not include a control condition, but the low support group will serve as a ‘usual practice’ comparator.
- Student-level data will only be collected in a sub-set of schools by secondary teachers, which may compromise internal validity.

For peer review only

For peer review only - http://bmjopen.bmj.com/site/about/guidelines.xhtml

INTRODUCTION

Physical inactivity has been described as a global health issue and is the fourth leading cause of premature death worldwide[1]. The World Health Organisation (WHO) recommends children and adolescents to participate in 60 minutes of (predominantly aerobic) moderate-to-vigorous physical activity (MVPA) daily, as well as muscle strengthening activity at least three days per week[2]. Despite these recommendations, physical activity declines substantially throughout adolescence resulting in many adolescents failing to meet daily physical activity guidelines[3]. Fewer than 1 in 50 Australian adolescents (15 to 17 years old) meet MVPA and muscle strengthening activity recommendations[4]. Poor rates of muscle strengthening activity may explain the secular decline in muscular fitness (i.e., strength, power, and endurance) of Australian youth over the past 30 years[5] with a similar decline seen internationally[6, 7]. This suggests that youth participation in muscle strengthening activity may have declined globally. Muscular fitness is associated with various markers of health and well-being among children and adolescents in both cross-sectional[8] and prospective[9] studies. This prompted our team to investigate the key role of muscle strengthening activity during the school years.

Resistance training (RT) is a specialised form of muscle strengthening activity. It can be performed with or without equipment using a variety of resistive loads, and in a range of settings (e.g., at home, school, local park, or gym/fitness centre)[10]. When performed routinely, RT may lead to muscle hypertrophy (i.e., increase in muscle size), improved muscular fitness (i.e., muscle strength, power, and/or endurance), body composition (i.e., increases in fat free mass and reductions in fat mass), and mental health (i.e., self-esteem) in school-aged youth[11]. RT is also recommended within global physical activity guidelines for adults[1], as it benefits a wide range of physical and mental health outcomes (including risk of chronic disease and depression/anxiety). However, the performance of RT is often

For peer review only

perceived as more complex than aerobic activities (e.g., walking and jogging). This may explain why participation in RT is much lower than for MVPA during adulthood[2]. Gaining the knowledge, skills, and confidence to participate in RT sets children and adolescents up for participation in RT across the lifespan[12]. However, for decades a number of myths and misconceptions about the safety and appropriateness of strength training have prevented it from being offered to most school-aged youth[12]. Furthermore, among adults there are a number of reported barriers to participation in RT, including lack of confidence[13], low self-efficacy[13], lack of time[14], and perceived lack of access to necessary equipment[14]. These barriers may also be present for adolescents, as they likely lack the knowledge, skills and/or confidence to engage in RT despite their desire to try a broader range of physical activities[15]. It is therefore necessary that adolescents are provided with ample opportunities to develop the skills, knowledge, and confidence to engage in RT.

Schools are effective settings for health promotion as they provide access to most children and adolescents, and have the facilities, equipment and qualified staff needed to deliver interventions[16-18]. However, few school-based interventions provided adolescents with the confidence and competence to participate safely in RT[19] and no prior studies have done so at scale[19, 20]. 'Scale up' is defined as the 'deliberate effort to increase the impact of successfully tested health interventions to benefit more people'[21]. Few physical activity interventions have progressed beyond efficacy testing and no studies investigating scaled up RT interventions among secondary students appear within the extant literature[22]. The WHO has advised policy makers, funders, and researchers to focus their efforts on scaling up evidence-based physical activity programs whilst exploring various models of implementation[23]. Prior to investing in scale up, evaluating the effectiveness of models of implementation to inform future scale up is necessary to guide the efficient use of resources. 'Voltage drop' is associated with diminishing effectiveness when programs are implemented

For peer review only - http://bmjopen.bmj.com/site/about/guidelines.xhtml

at a larger scale[24, 25]. Evaluating different models of implementation will help to minimise this phenomenon.

Resistance Training for Teens (RT4T) is an evidence-based program designed to provide adolescents with foundational knowledge about RT and enhance competence, confidence, and motivation to engage in RT across the lifespan. It was also designed to align with the Australian Curriculum for Health and Physical Education to develop the movement skills and concepts that will enable students to participate in physical activities that contribute to their health and wellbeing[26]. We conducted a rigorous evaluation of the RT4T intervention using a cluster randomised controlled trial (RCT) in 16 schools (N = 607) between 2015-2016[27]. The intervention resulted in immediate and sustained improvements in upper body muscular fitness and RT skill competency, demonstrating an effective and scalable approach to delivering RT within secondary schools. During this period, we established a partnership with the NSW Department of Education to scale-up the program. With support from the NSW Department of Education, 468 teachers from 249 NSW Government schools were trained to deliver the program[27]. We estimate that the program reached ~10,000 students between August 2015 and October 2020. However, interviews conducted with a sample of teachers (N =19) who delivered RT4T, identified considerable variability in program delivery. Also, several barriers to implementation emerged that included lack of support, motivation and time[28] impacting on program fidelity and potential sustainability.

Implementation support typically used for school physical activity programs, is often criticised as being a ‘train and forget’ model. It comprises initial training workshops followed by limited ongoing support for schools. This approach is unlikely to address the primary impediments to program implementation but may explain some shortcomings in whether evidence-based health promotion programs are adopted, implemented, or maintained within schools. The most effective approach that optimises implementation fidelity and may sustain

For peer review only - http://bmjopen.bmj.com/site/about/guidelines.xhtml

efficacious programs over the longer-term remains unclear[29]. Therefore, the aim of this present trial is to evaluate the effects of three implementation support models on the reach, effectiveness, dose delivered, fidelity, adoption, sustainability, impact, and cost of RT4T.

METHODS

Experimental design

We will conduct a hybrid type III implementation-effectiveness trial design[30] to evaluate the implementation and effectiveness of the RT4T intervention. We aim to recruit 90 secondary schools and randomise them to one of three groups: (i) Low, (ii) Moderate, or (iii) High implementation support. The Low support group will act as a control group (usual practice). This is the professional development model traditionally used by the NSW Department of Education and the delivery model used for RT4T from 2015-2020. In Australia, the academic year is separated into four 'terms' of 10 weeks duration. Following training, teachers enrolled in our study will deliver the 8-week RT4T intervention within one (or more) of two school terms (i.e., within six months post-training). This provides teachers with some flexibility regarding the delivery period for RT4T within their academic year.

Regarding the timeline for the evaluation of the intervention (i.e., the aim of our study), study assessments are undertaken at baseline, 6-months and 18-months follow-up. Teachers will be encouraged to maintain delivering the program after our research has concluded. Our implementation trial is guided by expert recommendations[31] and is registered with the Australian New Zealand Clinical Trials Registry (ACTRN12622000861752). The design, conduct and reporting will adhere to the Consolidated Standards of Reporting Trials (CONSORT)[32] statement, the Standards for Reporting Implementation Studies (StaRI)[33] statement, and the Template for Intervention Description and Replication (TIDieR) checklist[34].

For peer review only - http://bmjopen.bmj.com/site/about/guidelines.xhtml

School recruitment and selection

Secondary schools (Government, Catholic and Independent) in five local health districts in NSW (Illawarra Shoalhaven, Northern NSW, South Eastern Sydney, Sydney, and Western NSW) will be eligible to participate in this study. Schools outside these regions will be able to attend the professional learning workshops and implement RT4T, but will not be included into the study to assess implementation outcomes. Eligible schools will be those that include students in Grades 9 and 10 (aged 14 to 16 years). We will use a range of evidence-based recruitment and retention strategies to maximise participation and minimise drop-out (at both school and teacher levels), including promotion within the NSW Department of Education School Sport Unit newsletter. This will include pre-notification, the use of a dedicated recruitment coordinator, repeated reminders, and deployment of staff from local health promotion teams to engage with schools.

Participants

Teachers: One or two teachers from each enrolled school that teaches students in Grade 9 and 10 will be recruited to act as a 'School Champion'. They will attend a single full-day professional learning workshop delivered by members of the research team with tertiary qualifications in physical education (PE), health promotion, exercise physiology and/or strength and conditioning. School Champions will receive curriculum materials and resources at the workshop and will be asked to deliver a separate 2-hour training for other Grade 9 and 10 teachers at their schools, who also have the option to implement the program in their classes. Teachers can deliver the RT4T sessions to their students during usual PE lessons, co-curricular school sport periods or within an elective subject known as Physical Activity and Sports Studies. Professional learning will align with the NSW Educational Standards

For peer review only - http://bmjopen.bmj.com/site/about/guidelines.xhtml

Authority accreditation process to incentivise teachers to participate. Lastly, the learning will contribute to teachers' required annual training hours on a yearly basis[35, 36].

Students: Secondary students in Grades 9 and 10 (aged 14 to 16 years) enrolled in the study schools will be eligible to participate. The target population has been selected as this is the period during which students start to drop out of organised sport and can benefit from exposure to lifelong physical activities [37] such as resistance training.

Primary and secondary outcomes

A range of quantitative and qualitative methods will be used to assess primary and secondary outcomes at baseline (T0), 6-months (T1) and 18-months follow-up (T2). Our primary outcome is reach, operationalised as the proportion of Grade 9 and 10 students from the study schools who participate in the RT4T program. Proportion will be calculated as a percentage of students from Grade 9 and 10 who participate in ≥50% of the RT4T practical sessions divided by the total number of students in Grade 9 and 10 at the study schools. Secondary outcome measures include, dose delivered, fidelity, sustainability, impact, cost effectiveness, and implementation determinants.

Sample size calculation

We aim to recruit 90 secondary schools, with 30 schools assigned to each of the three treatment arms. This will include an estimated 261 classes and *N*=~7,800 students across the three arms of the trial. Based on our previous research in secondary schools, we estimate 20% of schools will not provide usable data for our primary outcome at follow-up. As few studies have tested the effects of implementation strategies on the reach of the school-based physical activity interventions, there is little information to guide power calculations. Therefore, we estimated that the Low support group will achieve 12.5% reach, while the Moderate and High support groups will both achieve 25% reach (i.e., between group difference of 12.5%). Our

For peer review only - http://bmjopen.bmj.com/site/about/guidelines.xhtml

power calculation is based on 90% power, type 1 error rate of 0.025, and standard deviation of 12%. For the continuous secondary student effectiveness outcomes, we assumed an intraclass correlation coefficient (ICC) estimate of 0.2. If 10% of schools provide useable data (~780 students), our study will have 80% power to detect significant small-to-moderate effects between treatment arms.

Blinding and randomisation

After teachers have completed the RT4T workshop, schools will be randomised to one of three implementation support arms. Schools will be stratified by school type and location and randomised using a random number producing algorithm by an independent statistical analysis service. Data analysis will be conducted by individuals blinded to group allocation.

Evidence-based intervention

RT4T is an 8-week multi-component physical activity program, including practical and theory-based lessons designed to develop adolescents' knowledge, competence, confidence, and motivation to participate in muscle strengthening activity. The design and delivery of RT4T is guided by the Supportive, Active, Autonomous, Fair and Enjoyable (SAAFE) principles for organised physical activity sessions for school-aged youth[38]. The training sessions and progressions were revised based on the Youth Physical Development Centre Basic Resistance Training Curriculum developed by Radnor and colleagues[39].

The RT4T program includes four major components: (i) school-based RT sessions (delivered by teachers); (ii) theoretical classroom sessions (delivered by teachers); (iii) a smartphone phone and tablet app (used by students/ teachers) and (iv) energiser breaks (used by teacher during theoretical sessions).

School-based RT sessions

For peer review only - http://bmjopen.bmj.com/site/about/guidelines.xhtml

Teachers will deliver the RT4T program during their usual PE lessons, co-curricular or Physical Activity and Sports Studies. The RT4T practical sessions include four sub-components. Sub-components include: (i) movement based dynamic warm-up (5 minutes), (ii) RT skill development (GymFit: 15-20 minutes) focused on five RT movement categories (i.e., upper body push, upper body pull, lower body bilateral, lower body spilt, and core stability), (iii) choice of three different types of muscle strengthening activities: a fitness workout of the day (WOD), modified game with fitness infusion (GameFit), or a fun fitness challenge done to music (FunFit) (15-20 minutes); and (iv) cool down and static stretching (StretchFit: 5 minutes). Practical sessions will be supported using a variety of resources, including exercise cards (Figure 1) and the RT4T smartphone and tablet app (Figure 2). Teachers will be also provided with a handbook that includes all the practical activities and theoretical content.

Theoretical classroom-based lessons

Students will receive 8 x 40-minute theory-based lessons that accompany weekly practical lessons. Content is purposefully aligned with the Australian curriculum for Health and Physical Education (HPE)[40] and Australian Physical Literacy Framework[41]. The lesson topics are generally relevant to RT and adolescent health. Topics include: (i) introduction to RT, (ii) fitness, self-assessment, and self-monitoring, (iii) fitness and e-Health technologies, (iv) principles of training and RT program design, (v) nutrition and RT: myths and recommendations, (vi) social media, physical activity, and body image, (vii) yoga, Pilates and other community-based muscle strengthening activity; and (vii) reviewing fitness outcomes and realigning future goals.

Smartphone and tablet app

For peer review only

For peer review only - http://bmjopen.bmj.com/site/about/guidelines.xhtml

We have developed and updated IOS versions of the RT4T smartphone and tablet app to support teachers to implement the program (see Figure 2). Use of the app is optional and can be downloaded from the Apple app store on all IOS devices and will be utilised for the purpose of tracking fitness test data, providing options for workouts with visual demonstrations for exercise technique along with further resources. The app includes: (i) fitness testing and criterion-referenced standards, (ii) skill testing for evaluating RT skill competence, (iii) workouts pre-designed for random selection of bodyweight RT exercises organised by difficulty using a custom workout generator; and (iv) an exercise library enabling users to browse bodyweight exercises organised by the five RT movement categories.

Implementation strategies that support teachers to deliver RT4T

We will assess whether implementation strategies can be implemented effectively across three study arms that offer implementation support. We define implementation strategies as methods or techniques used to enhance adoption, implementation, or sustainability of the evidence-based intervention[42]. Study arms are (i) Low support or 'usual practice' (training and resources), (ii) Moderate support (training and resources + external support); and (iii) High support (training and resources + external support + equipment) (see Table 1). Implementation strategies were co-developed with our partners in the NSW Department of Education and NSW local health districts and guided by findings from our previous dissemination study[28]. Our strategies align with the taxonomy of strategies as described by Proctor et. al.[42].

Implementation framework and strategies

Our approach is guided by the Consolidated Framework for Implementation Research which describes constructs across five domains: (i) intervention characteristics (RT4T), (ii) outer

For peer review only - http://bmjopen.bmj.com/site/about/guidelines.xhtml

settings (NSW Department of Education), (iii) inner setting (government schools); and (iv) individual (student/teacher) characteristics, and (v) the process of implementation[43]. We describe relevant implementation strategies in Table 1. We categorised implementation strategies as per the Consolidated Framework for Implementation Research constructs and specified their usage using the Action, Actor, Context, Target, Time framework[44].

For peer review only

Table 1: Domains, constructs, and strategies used in our three implementation support models

Domains	Constructs	Strategies	Level of support		
			Low	Moderate	High
RT4T intervention	Evidence strength/quality	Findings from the RT4T cluster RCT were used in promotional and training materials.	✓	✓	✓
	Adaptability	Flexible intervention delivery model (PE, school sport or elective) requiring minimal access to equipment.	✓	✓	✓
	Design quality and packaging	Program resources developed by a professional graphic designer.	✓	✓	✓
Outer setting	Partnerships and investment	Investment from, and partnership with, key stakeholders from education (i.e., NSW Department of Education) and health (i.e., NSW Health local health districts).	✓	✓	✓
	External policy and incentives	Professional learning accreditation with national and state-based educational standards authorities. Alignment with state school physical activity policy.	✓	✓	✓
Inner setting	School culture	School Champions will run a 2-hour face-to-face professional development workshop for other teachers in their school on RT4T. Prior to this, the teachers will have completed a 30-minute pre-learning session on resistance training safety. In the 2-hour workshop the School Champion will:<br>● Present the evidence and the rationale of RT4T.<br>● Teachers will participate in RT4T activities, discuss the content, and gain consensus on how they will deliver RT4T.<br>● The School Champion will share and explain the resources they received at the School Champion workshop.	✓	✓	✓
	Leadership engagement	School Champions and Project Officers will meet with the school principal to ensure commitment. In this meeting they will:<br>● Show the 4-minute promo video which explains the benefits of RT, evidence of RT4T and an overview of the project.<br>● Discuss how RT4T will be delivered in the school, who is involved, the contact person, time commitment and the costs.<br>● Complete the teacher (from all teachers who will deliver RT4T) and principal informed consents and return to the research team by sending to: RTforTeens@newcaste.edu.au		✓	✓
	Resources	Schools will receive exercise technique and workout resources. These will be provided to the School Champion at the teacher workshop. All materials will also be available on a website where teacher can upload all the materials they need to deliver the program.	✓	✓	✓

For peer review only - http://bmjopen.bmj.com/site/about/guidelines.xhtml

	Equipment	Schools will be provided with a basic RT equipment pack (~$1,000 AUD). This includes 4x 5kg weighted bars, 4 x 3kg dumbbells, 2 x portable pull up bar stands, and 4 x sets of resistance bands. These will be delivered to the schools in the High support group following randomisation after the workshop.			✓
**Individuals**	Competence, opportunity, motivation<br>(teacher)	A full-day professional development workshop will be provided to School Champions. This workshop will be accredited with the NSW Educational Standards Authority and counts towards teachers professional learning. This will be delivered by the research team (UoN) who are experts in resistance training and school interventions. These will be held for groups of schools in the local area convenient to their location. Teacher relief for the School Champions will be provided by the School Sport Unit to the school to cover the cost of their attendance. Prior to attending the workshop, School Champions will complete the 30-minute online pre-learning on resistance training safety. The full-day workshop training will cover the following:<br>● Rationale for RT4T (i.e., prevalence of physical inactivity in youth, benefits of RT, and myths/facts regarding participation in RT).<br>● Adaptability of RT4T.<br>● Skills and demonstrations of the movements.<br>● RT4T lesson content and alignment with the Personal Development, Health, and Physical Education syllabus.<br>● Roles and responsibilities of School Champions, teachers, and Project Officers.<br>● Introduction to the RT4T app.	✓	✓	✓
	Perceived barriers (student)	Addresses student motivation using SAAFE principles.<br>● Teachers will learn to deliver RT4T using the SAAFE principles.	✓	✓	✓
**Implementation process**	Planning for implementation	Teachers will complete an action plan template to support implementation of RT4T in their school. The action plan will include:<br>● When they are going to train their staff and how.<br>● When they are going to get resources to teachers and how.<br>● Roles and responsibilities of the School Champion and teachers.<br>● School Champions will complete the action plan during the full-day professional development workshop.	✓	✓	✓
	Project Officers	School Champions will be connected to a Project Officer (i.e., Project Officers from five local health district) who will provide ongoing support to the School Champions after they have attended the workshop. The Project Officer will:<br>● Attend the in person principal meeting with the School Champion to gain principal commitment (only if this is not done before workshop already).<br>● Email/phone/visit the School Champions.<br>● Complete the checklist developed by the research team.		✓	✓

For peer review only - http://bmjopen.bmj.com/site/about/guidelines.xhtml

		• Stay in touch with the project manager from RT4T and provide all the needed details that are requested by the research team (e.g., keep track of the support provided).			
	Evaluation and feedback	The Project Officers will observe approximately two RT4T session per school and provide feedback on the RT4T structure via email.<br>• Observations will be conducted using a standardised checklist developed by the research team that assess aspects of the RT4T session (e.g., did the teachers conduct the warm-up?).<br>• Project Officers will only provide feedback on the RT4T structure via email.		✓	✓

345 *Note.* RT4T = Resistance Training for Teens; SAAFE = supportive, active, autonomous, fair, and enjoyable

For peer review only

Professional learning for teachers

The School Champion will complete a professional 30-minute pre-learning online course prior to program delivery. This will include modules covering foundational principles of RT and specific safety recommendations to deliver RT to school-aged youth. Following initial completion of the online learning, the School Champion will attend a single full-day workshop covering the background/rationale, structure, and implementation of the RT4T program, delivered by members of the research team with relevant expertise in physical education, health promotion, exercise physiology, and strength and conditioning.

School Champions will be asked to attend the professional learning workshop and deliver a compressed training module to other Grade 9 and 10 teachers within their schools. The compressed module comprises a train-the-trainer approach that is supported by resources (i.e., presentation slides) provided by the research team. Our goal is to enable all interested and eligible teachers to deliver the RT4T program to maximise the reach of the RT4T program across the eligible student population.

Project Officers

The Project Officers (i.e., Project Officers from five local health districts) will participate in a full-day training delivered by the research team. The training will be focused on the following: (i) RT4T program content, (ii) strategies to support teachers, (iii) Project Officer roles and responsibilities, and (iv) how to assess fidelity of the RT4T program. Following the teacher workshop, schools randomised to the Moderate and High support groups will receive external support from Project Officers). Project Officers with relevant qualifications in physical education, health promotion, exercise physiology, and/or strength and conditioning will observe teachers' implementation of the RT4T practical sessions, provide ongoing support, and address the various barriers to program implementation. School Champions and

For peer review only - http://bmjopen.bmj.com/site/about/guidelines.xhtml

Project Officers in the Moderate and High support groups will meet with school leaders (principal or head of department) to ensure commitment to the RT4T program.

Materials and equipment

All participating schools will receive 100 hardcopy exercise cards to use during practical sessions (see Figure 1). Schools allocated to the High implementation support group will also receive a basic RT equipment pack. Each equipment pack will include a variety of low cost, versatile exercise equipment (e.g., resistance bands,3kg dumbbells and 5kg weighted bar).

Measures and data collection

Quantitative assessments will be conducted at baseline (T0), 6-months (post intervention; T1) and 18-months (1 year post intervention; T2). Teachers will complete an online survey (T0, T1 and T2). Personal Development, Health, and Physical Education Heads of Department (N=18) and School Champions (N=18) will be invited to participate in face-to-face or virtual interviews (T1 and T2).

Implementation evaluation

Primary implementation outcome

We define an implementation outcome as the intentional actions to deliver a policy or an intervention[45, 46].

Reach: Our primary outcome is reach, operationalised as the proportion of Grade 9 and 10 students from the study schools who participate in the RT4T program. Proportion will be calculated as a percentage of students from Grade 9 and 10 who participate in ≥50% of the RT4T practical sessions divided by the total number of students in Grade 9 and 10 a. We will assess reach by collecting teacher logbooks (i.e., attendance lists) at T1 and T2. We have used logbooks previously in school-based health promotion trials[47, 48] and achieved high

For peer review only - http://bmjopen.bmj.com/site/about/guidelines.xhtml

completion rates, good validity, and reliability[49]. A research team member will request that School Champions provide a copy of teacher logbooks that describe details of the RT4T program. We will collect representativeness data [(i.e., School Index of Community Socio-Educational Advantage value, indigenous students (%), and students with language backgrounds other than English (%)] at the school level using the My School website. This information will be reported descriptively.

Secondary implementation outcomes

Dose delivered: Dose delivered is the number of RT4T practical and theory lessons delivered to students. Teachers will be asked to record this information in the RT4T teacher handbooks.

Fidelity*:* Project Officers will observe approximately two RT4T practical sessions delivered by a teacher per school (Moderate and High support arms) (total observations N = ~120). The research team will observe approximately one RT4T practical session per school allocated to the Low support arm (total observations N = ~30). Session fidelity will be assessed by completing an observation checklist that describes the RT4T structure and process (e.g., did the teacher include a warm-up?) and to what extent they implemented the SAAFE principles (e.g., teacher was supportive and promoted positive student interactions, with answer options ranging from 1 = '*Strongly disagree'* to 4 = '*Strongly agree'*).

Sustainability*:* We define sustainability as the extent to which the program is embedded within school practices. It will be assessed in two ways. First, through an interview with the Personal Development, Health, and Physical Education, Head of Department (at T2). Second, School Champions will be asked to complete the Provider Report of Sustainment Scale (PRESS) sustainability tool[50]. The tool is a brief, pragmatic and generalisable 3-item measure for frontline service providers. It assesses evidenced based practice in different settings and has been shown to be a valid measure of sustainment.

For peer review only - http://bmjopen.bmj.com/site/about/guidelines.xhtml

Implementation determinants: We define implementation determinants as factors believed or empirically shown to influence implementation outcomes[51]. We will assess the following determinants: culture, acceptability, feasibility, adaptability, compatibility (appropriateness), dose (satisfaction), capabilities, opportunities, and motivations (COM-B) using a teacher survey (~20-minutes). This survey has not been used previously, however is based upon a body of literature per different implementation outcomes including (n = number of survey items): participation in muscle strengthening exercise (6)[52] culture (1)[53], acceptability (1), feasibility (1), compatibility (appropriateness)(1) [54], adaptability (3), dose (satisfaction) (2)[55], capability (4), opportunity (5), and motivation (4)[56]. All School Champions will complete the questionnaire and will advise other teachers delivering the program within their school to do the same. The research team will send reminders for survey completion to all teachers at 6-month and 18-month follow-up.

Impact evaluation

Student-level outcome data will be collected from a convenience sample of students who provide informed parental consent. We anticipate 10% of the sample will provide usable data (N~780 students). Fitness testing, participation in muscle strengthening activity and RT skill competency will be assessed using the RT4T app. The tablet version of the app (adapted from the 'Burn 2 Learn' program)[57] will be used by teachers to assess students' fitness, student's participation in muscle strengthening activity and RT skill competency. We will evaluate upper body muscular endurance using the 90-degree push-up test[58]. Isometric abdominal muscular endurance will be measured using the plank hold test[59]. Standing long jump will assess muscular power, 1-minute sit to stand will assess lower body muscular endurance and the 10 x 5m shuttle run will assess speed and agility. These fitness assessments are integrated into the RT4T program and conducted by the teachers at the start (week 1) and at the end (week 8) of the 8-week intervention period.

For peer review only - http://bmjopen.bmj.com/site/about/guidelines.xhtml

Statistical analysis

Statistical analyses will be conducted by an independent statistical analysis service - Clinical Research Design, Information Technology and Statistical Support from Hunter Medical Research Institute. Analyses of the primary (program reach) and secondary outcomes will be conducted using logistic mixed models (SAS Institute Inc, Cary, NC) for dichotomous outcomes, and ordinal or linear mixed effects regression models for continuous outcomes. The primary outcome (i.e., Reach) will be collected at the school level and assessed using t-tests. Alpha levels will be set at $p<0.025$ for the comparisons of the Moderate and High support arms against the Low support arm. If the p-values for the differences between the Moderate and High support arms with the Low support arm reach this threshold, the Moderate and High support arms will be compared at a 5% significance threshold. For student level outcomes, statistical analyses will be adjusted for the clustering of effects at the class level, as students from each school are nested in classes. Although clustering at the school level is negligible after adjusting for clustering at the class level, we will test this assumption and adjust our analyses for school-level clustering if required. For teacher-level outcomes, clustering will be accounted for at the school-level. Three potential moderators of effects will be explored using interaction terms (i.e., type of Project Officer, socio-economic status of school, and school location). School socio-economic status will be determined using the Index of Community Socio-Educational Advantage (ICSEA), which will be obtained from the MySchool website[60]. School ICSEA values are determined using student-level parent occupation and education data, school location (i.e., remoteness), and percentage of indigenous student enrolment.

Data Monitoring

For peer review only - http://bmjopen.bmj.com/site/about/guidelines.xhtml

All entered data will be deidentified using participant codes and will be stored electronically within the RT4T app, and in a password protected drive at the University of Newcastle. Data will be checked for implausible values, and 20% of the data will be entered twice to confirm accuracy. It is not expected that participants will be any greater risk of adverse events than they would be when participating in other types of school-based physical activity. However, the teacher handbook includes a section for teachers to report any adverse events that may occur. Any adverse events will be documented and reported to the relevant ethics committee. Any amendments to the study protocols will be publicly available via the Australian and New Zealand Clinical Trials Registry (trial number: ACTRN12622000861752).

Economic evaluation

The economic evaluation will assess the costs and consequences of the RT4T program across the three trial arms and will also include a budget impact analysis. The results of the cost-consequence analysis are presented as a scorecard comprising the total incremental cost of delivering the intervention alongside the range of outcomes reflected in the primary and secondary trial outcomes (consequences). This approach allows decision makers to interpret the costs and outcomes of an intervention in a way that is relevant to their decision-making context.

Costs comprise the resource use associated with the intervention and implementation, while research costs are excluded. The opportunity cost for teacher and staff time will be prospectively measured and valued using pro-rata Department of Education salary levels. The implicit cost of the spaces used to conduct the intervention will also be reported, as well as equipment costs. The budget impact analysis will be conducted to estimate the cost of scaling up the intervention across NSW and Australia. Consideration will be given to any cost offsets

For peer review only - http://bmjopen.bmj.com/site/about/guidelines.xhtml

which would result from the scaling up of the intervention. **ETHICS AND DISSEMINATION**

Ethics approval has been obtained from the University of Newcastle (H-2021-0418), the NSW Department of Education (SERAP:2022215), Hunter New England Human Research Ethics Committee (2023/ETH00052), and the Catholic Schools Office. Students attending schools where the program is delivered within the five local health districts will require parental consent for their fitness data to be recorded within the RT4T app. Students attending schools outside the five indicated local health districts will need to provide opt-out consent if they do not want their fitness data collected within the RT4T app. Our findings will be published in open access peer-reviewed journals. We will provide the NSW Department of Education, NSW Ministry of Health, and all participating schools with a detailed report of our study findings. We will support on-going dissemination of RT4T in NSW and beyond via a series of professional learning workshops.

DISCUSSION

Few school-based interventions provide adolescents with the confidence and competence to participate safely in RT and no prior studies have done so at scale[19]. RT4T is an evidence-based program designed to provide adolescents with the competence, confidence, knowledge, and motivation to engage in RT throughout their lifetime. Importantly, RT4T was developed with scale-up in mind using the Consolidated Framework for Implementation Research (CFIR) to guide implementation.

Our study fills an important gap regarding the scale-up of effective school-based physical activity interventions[61]. Despite the large number of school-based efficacy and effectiveness trials, there is still only a small number of dissemination and implementation trials that have been conducted. As such, little is known regarding the strategies to support

For peer review only

the implementation of physical activity programs in schools. While the health benefits are well-established, there is a lack of school-based interventions supporting adolescents' safe participation in structured RT. This research will have widespread population health benefits as we leverage relationships through partnerships with local health district Project Officers and schools to increase program scale-up.

However, there are limitations that should be noted. First, our study does not include a control group. However, the low implementation support group will serve as the RT4T 'usual practice' comparator. Second, our expanded delivery method is reliant upon a 'train-the-trainer' model, and increasing the number of schools involved will help to determine feasibility across varying school contexts. Considering this, there is a risk of compromised fidelity to the intervention. Finally, regarding the effectiveness evaluation, there is a risk of measurement bias influencing internal validity with only a sub-set of schools providing student-level data, which is to be collected by secondary teachers.

Contributors: HTK: software, resources, writing - initial draft, writing - review and editing. JJS: conceptualisation, methodology, software, resources, writing – initial draft, writing - review and editing, supervision and funding acquisition. AV: methodology, software, resources, writing – initial draft, writing – review and editing, supervision, project administration. SGK: conceptualisation, methodology, resources, writing – original draft, writing – review and editing, funding acquisition. JS: conceptualisation, methodology, resources, writing – original draft, writing – review and editing, funding acquisition. HM: conceptualisation, methodology, software, writing – original draft, writing – review and editing, funding acquisition. NN: conceptualisation, methodology, writing – original draft, writing – review and editing, funding acquisition. RS: conceptualisation, methodology, writing – original draft, writing – review and editing, funding acquisition. PJM: conceptualisation, methodology, writing – original draft, writing – review and editing,

For peer review only - http://bmjopen.bmj.com/site/about/guidelines.xhtml

funding acquisition. JS: conceptualisation, methodology, writing – original draft, writing – review and editing, funding acquisition. DP: conceptualisation, methodology, writing – original draft, writing – review and editing, funding acquisition. JB: conceptualisation, methodology, writing – review and editing, funding acquisition. RSL: methodology, resources, writing – review and editing. CO: methodology, writing – review and editing. PR: methodology, review and editing. KP: methodology, resources, writing – review and editing. MH: writing – review and editing, project administration. SL: writing – review and editing, project administration. JN: writing – review and editing, project administration. AV: writing – review and editing, project administration. AZ: writing – review and editing, project administration. DRL: conceptualisation, methodology, software, resources, writing – original draft, writing – review and editing, supervision and funding acquisition.

Funding: This project is funded by a National Health and Medical Research Council (NHMRC) Partnership Grant (APP2010866). DRL is funded an NHMRC Senior Research Fellowship (APP1154507). NN is funded by the Medical Research Future Fund (MRFF) Investigator Grant (GS2000053). This project is supported and co-designed with the NSW Department of Education.

Disclaimer: The study funders will have no role in data collection, analysis, interpretation, or writing nor will they have influence over the publication of findings.

Competing interests: None declared.

Patient and public involvement: The NSW Department of Health, NSW local health districts, secondary school teachers, and students were involved in the design of the RT4T intervention.

Patient consent for publication: Not applicable.

Provenance and peer review: Not commissioned; externally peer reviewed.

For peer review only - http://bmjopen.bmj.com/site/about/guidelines.xhtml

561 **Supplemental material:** Not applicable.

562 **Open access:** This is an open access article distributed in accordance with the Creative
563 Commons Attribution 4.0 Unported (CC BY 4.0) license, which permits others to copy,
564 redistribute, remix, transform and build upon this work for any purpose, provided the original
565 work is properly cited, a link to the licence is given, and indication of whether changes were
566 made. See: https://creativecommons.org/ licenses/by/4.0/

567 **ORCID IDs:**

568 Hayden T Kelly http://orcid.org/0000-0002-4533-6945

569 Angeliek Verdonschot https://orcid.org/0000-0002-1519-3402

570 Sarah Kennedy https://orcid.org/0000-0001-9804-616X

571 Nicole Nathan https://orcid.org/0000-0002-7726-1714

572 Philip Morgan https://orcid.org/0000-0002-5632-8529

573 Jo Salmon https://orcid.org/0000-0002-4734-6354

574 Dawn Penney https://orcid.org/0000-0002-2000-8953

575 James Boyer https://orcid.org/0000-0002-0585-4238

576 Rhodri Lloyd https://orcid.org/0000-0001-8560-1566

577 Jennifer Norman https://orcid.org/0000-0001-6344-2605

578 Alexander Voukelatos https://orcid.org/0000-0003-3131-761X

579 Avigdor Zask https://orcid.org/0000-0002-3888-7335

580 David R Lubans http://orcid.org/0000-0002-0204-8257

581 **References**

For peer review only

1. Kohl, H.W., et al., *The pandemic of physical inactivity: global action for public health.* The lancet, 2012. **380**(9838): p. 294-305.
2. Bull, F.C., et al., *World Health Organization 2020 guidelines on physical activity and sedentary behaviour.* British journal of sports medicine, 2020. **54**(24): p. 1451-1462.
3. Farooq, M.A., et al., *Timing of the decline in physical activity in childhood and adolescence: Gateshead Millennium Cohort Study.* British journal of sports medicine, 2018. **52**(15): p. 1002-1006.
4. Statistics, A.B.o., *National Health Survey: first results, 2017–18*. 2019, Australian Bureau of Statistics Canberra.
5. Fraser, B.J., et al., *The great leap backward: Changes in the jumping performance of Australian children aged 11− 12-years between 1985 and 2015.* Journal of sports sciences, 2019. **37**(7): p. 748-754.
6. Tomkinson, G.R., et al., *Temporal trends in the standing broad jump performance of 10,940,801 children and adolescents between 1960 and 2017.* Sports medicine, 2021. **51**: p. 531-548.
7. Ortega, F.B., et al., *European fitness landscape for children and adolescents: updated reference values, fitness maps and country rankings based on nearly 8 million test results from 34 countries gathered by the FitBack network.* British journal of sports medicine, 2023. **57**(5): p. 299-310.
8. Smith, J.J., et al., *The health benefits of muscular fitness for children and adolescents: a systematic review and meta-analysis.* Sports medicine, 2014. **44**: p. 1209-1223.
9. García-Hermoso, A., R. Ramírez-Campillo, and M. Izquierdo, *Is muscular fitness associated with future health benefits in children and adolescents? A systematic review and meta-analysis of longitudinal studies.* Sports Medicine, 2019. **49**: p. 1079-1094.
10. Lloyd, R.S., et al., *Position statement on youth resistance training: the 2014 International Consensus.* British journal of sports medicine, 2014. **48**(7): p. 498-505.
11. Brusseau, T., S. Fairclough, and D. Lubans, *The Routledge handbook of youth physical activity*. 2020: Routledge.
12. Faigenbaum, A.D., et al., *Mythology of youth resistance training.* British Journal of Sports Medicine, 2022.
13. Rhodes, R.E., et al., *Factors associated with participation in resistance training: a systematic review.* British Journal of Sports Medicine, 2017. **51**(20): p. 1466-1472.
14. Steele, J., et al., *A higher effort-based paradigm in physical activity and exercise for public health: making the case for a greater emphasis on resistance training.* BMC public health, 2017. **17**(1): p. 1-8.
15. Tannehill, D., et al., *What young people say about physical activity: the Children's Sport Participation and Physical Activity (CSPPA) study.* Sport, Education and Society, 2015. **20**(4): p. 442-462.
16. Hills, A.P., D.R. Dengel, and D.R. Lubans, *Supporting public health priorities: recommendations for physical education and physical activity promotion in schools.* Progress in cardiovascular diseases, 2015. **57**(4): p. 368-374.
17. Poland, B., G. Krupa, and D. McCall, *Settings for health promotion: an analytic framework to guide intervention design and implementation.* Health promotion practice, 2009. **10**(4): p. 505-516.
18. Neufeld, J. and J. Kettner, *The settings approach in public health: thinking about schools in infectious disease prevention and control.* National collaborative center of infectious diseases: University of Manitoba, 2014.
19. Cox, A., et al., *Efficacy of school-based interventions for improving muscular fitness outcomes in adolescent boys: A systematic review and meta-analysis.* Sports Medicine, 2020. **50**: p. 543-560.
20. Malina, R.M., *Weight training in youth-growth, maturation, and safety: an evidence-based review.* Clinical journal of sport medicine, 2006. **16**(6): p. 478-487.
21. Milat, A.J., et al., *A guide to scaling up population health interventions.* Public health research & practice, 2016.

For peer review only

For peer review only - http://bmjopen.bmj.com/site/about/guidelines.xhtml

22. Reis, R.S., et al., *Scaling up physical activity interventions worldwide: stepping up to larger and smarter approaches to get people moving.* The lancet, 2016. **388**(10051): p. 1337-1348.
23. Organization, W.H., *Nine steps for developing a scaling-up strategy*. 2010: World Health Organization.
24. Chambers, D.A., R.E. Glasgow, and K.C. Stange, *The dynamic sustainability framework: addressing the paradox of sustainment amid ongoing change.* Implementation science, 2013. **8**(1): p. 1-11.
25. McCrabb, S., et al., *Scaling-up evidence-based obesity interventions: a systematic review assessing intervention adaptations and effectiveness and quantifying the scale-up penalty.* Obesity Reviews, 2019. **20**(7): p. 964-982.
26. *Rationale*, in *The Australian Curriculum*.
27. Kennedy, S.G., et al., *Implementing resistance training in secondary schools: a cluster randomized controlled trial.* Medicine & Science in Sports & Exercise, 2018. **50**(1): p. 62-72.
28. Kennedy, S.G., et al., *Implementing resistance training in secondary schools: an exploration of teachers' perceptions.* Translational Journal of the American College of Sports Medicine, 2018. **3**(12): p. 85-96.
29. Shoesmith, A., et al., *Barriers and facilitators influencing the sustainment of health behaviour interventions in schools and childcare services: a systematic review.* Implementation Science, 2021. **16**(1): p. 62.
30. Landes, S.J., S.A. McBain, and G.M. Curran, *Reprint of: an introduction to effectiveness-implementation hybrid designs.* Psychiatry research, 2020. **283**: p. 112630.
31. McKay, H., et al., *Implementation and scale-up of physical activity and behavioural nutrition interventions: an evaluation roadmap.* International Journal of Behavioral Nutrition and Physical Activity, 2019. **16**(1): p. 1-12.
32. Health, D.o., *Australia's Long Term National Health Plan.*, C.A. Government, Editor. 2019.
33. Pinnock, H., et al., *Standards for reporting implementation studies (StaRI) statement.* bmj, 2017. **356**.
34. Wolfenden, L., et al., *Obtaining active parental consent for school-based research: a guide for researchers.* Australian and New Zealand journal of public health, 2009. **33**(3): p. 270-275.
35. Kennedy, S.G., et al., *Implementation at-scale of school-based physical activity interventions: A systematic review utilizing the RE-AIM framework.* Obesity Reviews, 2021. **22**(7): p. e13184.
36. Sutherland, R.L., et al., *The physical activity 4 everyone cluster randomized trial: 2-year outcomes of a school physical activity intervention among adolescents.* American journal of preventive medicine, 2016. **51**(2): p. 195-205.
37. Hulteen, R.M., et al., *Development of foundational movement skills: A conceptual model for physical activity across the lifespan.* Sports medicine, 2018. **48**: p. 1533-1540.
38. Lubans, D.R., et al., *Framework for the design and delivery of organized physical activity sessions for children and adolescents: rationale and description of the 'SAAFE' teaching principles.* International journal of behavioral nutrition and physical activity, 2017. **14**(1): p. 1-11.
39. Radnor, J.M., et al., *Developing athletic motor skill competencies in youth.* Strength & Conditioning Journal, 2020. **42**(6): p. 54-70.
40. Australian Curriculum, A.a.R.A., *ACARA - Curriculum*. 2022.
41. Keegan R, B.L., Dudley D., *The Physical Literacy Framework*, S. Australia, Editor. 2019.
42. Proctor, E.K., B.J. Powell, and J.C. McMillen, *Implementation strategies: recommendations for specifying and reporting.* Implementation science, 2013. **8**(1): p. 1-11.
43. Keith, R.E., et al., *Using the Consolidated Framework for Implementation Research (CFIR) to produce actionable findings: a rapid-cycle evaluation approach to improving implementation.* Implementation Science, 2017. **12**(1): p. 1-12.
44. Presseau, J., et al., *Action, actor, context, target, time (AACTT): a framework for specifying behaviour.* Implementation Science, 2019. **14**: p. 1-13.

For peer review only - http://bmjopen.bmj.com/site/about/guidelines.xhtml

45. Proctor, E., et al., *Outcomes for implementation research: conceptual distinctions, measurement challenges, and research agenda.* Administration and policy in mental health and mental health services research, 2011. **38**: p. 65-76.
46. Peters, D.H., et al., *Implementation research: what it is and how to do it.* Bmj, 2013. **347**.
47. Nathan, N., et al., *Multi-strategy intervention increases school implementation and maintenance of a mandatory physical activity policy: outcomes of a cluster randomised controlled trial.* British Journal of Sports Medicine, 2022. **56**(7): p. 385-393.
48. Nathan, N.K., et al., *Implementation of a school physical activity policy improves student physical activity levels: outcomes of a cluster-randomized controlled trial.* Journal of Physical Activity and Health, 2020. **17**(10): p. 1009-1018.
49. Cradock, A.L., et al., *Impact of the Boston active school day policy to promote physical activity among children.* American Journal of Health Promotion, 2014. **28**(3_suppl): p. S54-S64.
50. Moullin, J.C., et al., *Provider REport of Sustainment Scale (PRESS): development and validation of a brief measure of inner context sustainment.* Implementation Science, 2021. **16**(1): p. 1-10.
51. Nilsen, P. and S. Bernhardsson, *Context matters in implementation science: a scoping review of determinant frameworks that describe contextual determinants for implementation outcomes.* BMC health services research, 2019. **19**(1): p. 1-21.
52. Shakespear-Druery, J., et al., *Muscle-Strengthening Exercise Questionnaire (MSEQ): an assessment of concurrent validity and test–retest reliability.* BMJ Open Sport & Exercise Medicine, 2022. **8**(1): p. e001225.
53. Carlson, J.A., et al., *Contextual factors related to implementation of classroom physical activity breaks.* Translational behavioral medicine, 2017. **7**(3): p. 581-592.
54. Weiner, B.J., et al., *Psychometric assessment of three newly developed implementation outcome measures.* Implementation science, 2017. **12**: p. 1-12.
55. Kennedy, S.G., et al., *Process evaluation of a school-based high-intensity interval training program for older adolescents: the burn 2 learn cluster randomised controlled trial.* Children, 2020. **7**(12): p. 299.
56. Keyworth, C., et al., *Acceptability, reliability, and validity of a brief measure of capabilities, opportunities, and motivations ("COM-B").* British journal of health psychology, 2020. **25**(3): p. 474-501.
57. Lubans, D.R., et al., *Time-efficient intervention to improve older adolescents' cardiorespiratory fitness: findings from the 'Burn 2 Learn' cluster randomised controlled trial.* British journal of sports medicine, 2021. **55**(13): p. 751-758.
58. Lubans, D.R., et al., *Test–retest reliability of a battery of field-based health-related fitness measures for adolescents.* Journal of sports sciences, 2011. **29**(7): p. 685-693.
59. Boyer, C., et al., *Feasibility, validity, and reliability of the plank isometric hold as a field-based assessment of torso muscular endurance for children 8–12 years of age.* Pediatric exercise science, 2013. **25**(3): p. 407-422.
60. Australian Curriculum, A.a.R.A. *Guide to understanding the Index of Community Socioeducational Advantage (ICSEA)*. My School 2020; Available from: https://www.myschool.edu.au/media/1820/guide-to-understanding-icsea-values.pdf.
61. Kennedy, S.G., et al., *Implementation and scale-up of school-based physical activity interventions*, in *The routledge handbook of youth physical activity*. 2020, Routledge. p. 438-460.

For peer review only

For peer review only - http://bmjopen.bmj.com/site/about/guidelines.xhtml

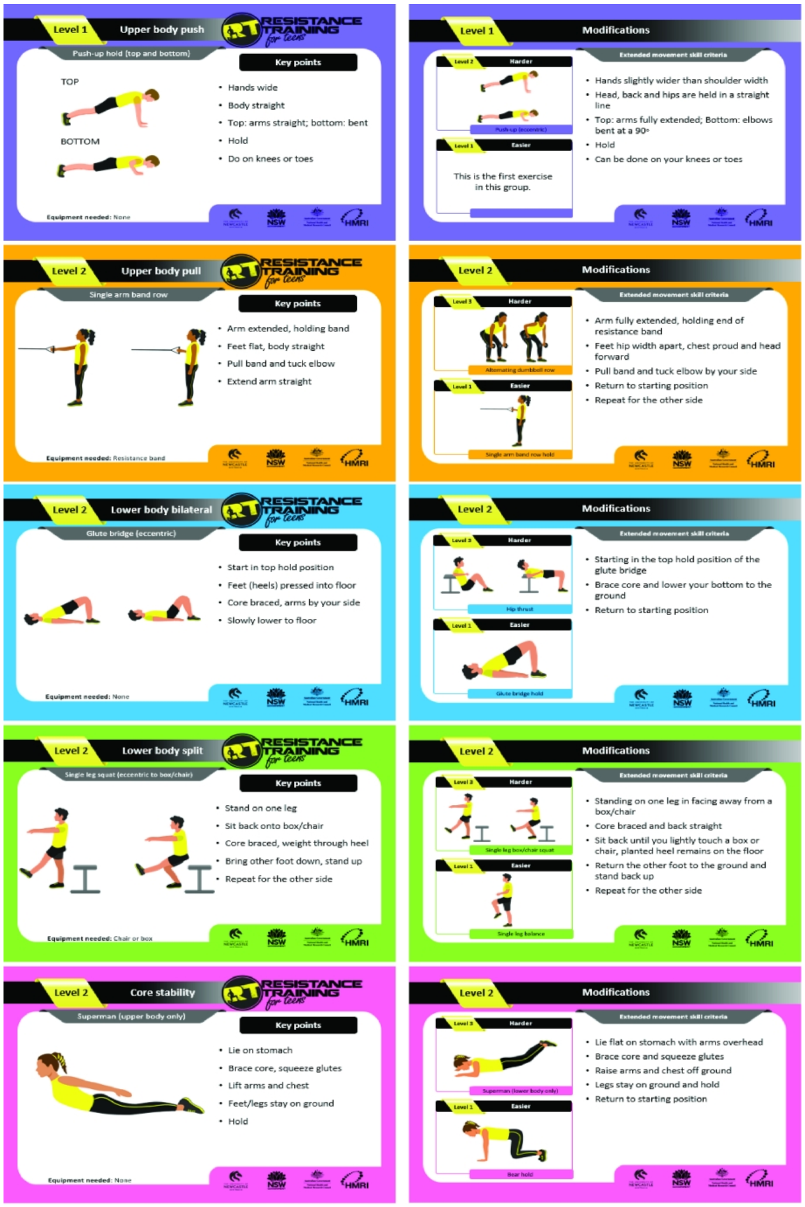

197x297mm (300 x 300 DPI)

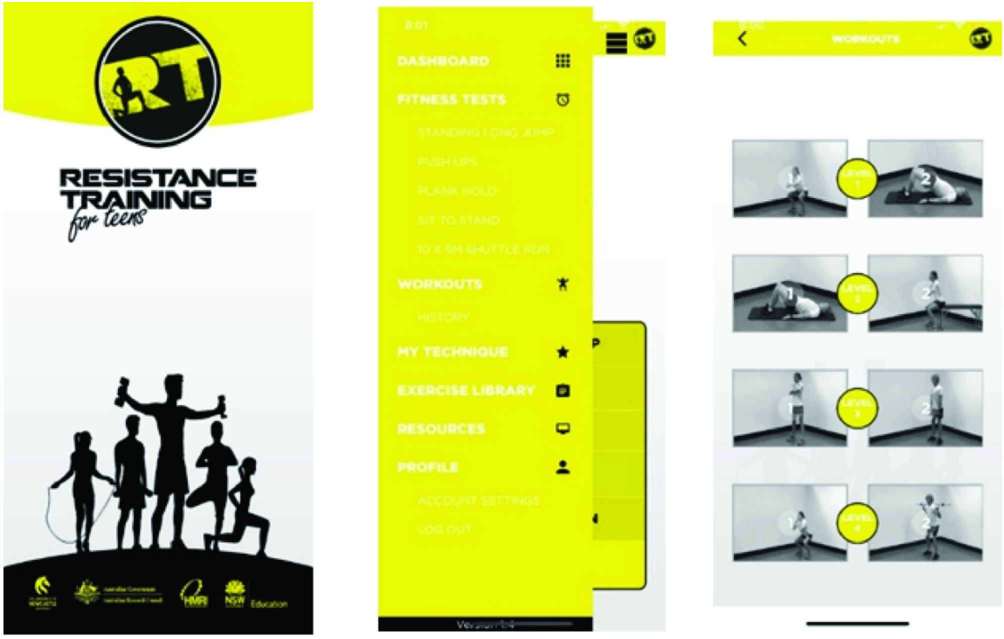

214x135mm (300 x 300 DPI)

TIDieR Template for Intervention Description and Replication

The TIDieR (Template for Intervention Description and Replication) Checklist*:

Information to include when describing an intervention and the location of the information

Item number	Item	Where located ** Primary paper (page or appendix number)	Other † (details)
	**BRIEF NAME**		
**1.**	Provide the name or a phrase that describes the intervention.	page 2	______________
	**WHY**		
**2.**	Describe any rationale, theory, or goal of the elements essential to the intervention.	pages 7-10	______________
	**WHAT**		
**3.**	Materials: Describe any physical or informational materials used in the intervention, including those provided to participants or used in intervention delivery or in training of intervention providers. Provide information on where the materials can be accessed (e.g. online appendix, URL).	pages 10-11	______________
**4.**	Procedures: Describe each of the procedures, activities, and/or processes used in the intervention, including any enabling or support activities.	pages 10-13	______________
	**WHO PROVIDED**		
**5.**	For each category of intervention provider (e.g. psychologist, nursing assistant), describe their expertise, background and any specific training given.	pages 11,20,21	______________
	**HOW**		
**6.**	Describe the modes of delivery (e.g. face-to-face or by some other mechanism, such as internet or telephone) of the intervention and whether it was provided individually or in a group.	pages 13-14	______________
	**WHERE**		
**7.**	Describe the type(s) of location(s) where the intervention occurred, including any necessary infrastructure or relevant features.	pages 13-14	______________

For peer review only - http://bmjopen.bmj.com/site/about/guidelines.xhtml

For peer review only

	**WHEN and HOW MUCH**		
**8.**	Describe the number of times the intervention was delivered and over what period of time including the number of sessions, their schedule, and their duration, intensity or dose.	pages 13-14	______________
	**TAILORING**		
**9.**	If the intervention was planned to be personalised, titrated or adapted, then describe what, why, when, and how.	pages 13-15	______________
	**MODIFICATIONS**		
**10.‡**	If the intervention was modified during the course of the study, describe the changes (what, why, when, and how).	N/A	______________
	**HOW WELL**		
**11.**	Planned: If intervention adherence or fidelity was assessed, describe how and by whom, and if any strategies were used to maintain or improve fidelity, describe them.	pages 21-22	______________
**12.‡**	Actual: If intervention adherence or fidelity was assessed, describe the extent to which the intervention was delivered as planned.	N/A	______________

** **Authors** - use N/A if an item is not applicable for the intervention being described. **Reviewers** – use '?' if information about the element is not reported/not sufficiently reported.

† If the information is not provided in the primary paper, give details of where this information is available. This may include locations such as a published protocol or other published papers (provide citation details) or a website (provide the URL).
‡ If completing the TIDieR checklist for a protocol, these items are not relevant to the protocol and cannot be described until the study is complete.

* We strongly recommend using this checklist in conjunction with the TIDieR guide (see *BMJ* 2014;348:g1687) which contains an explanation and elaboration for each item.

* The focus of TIDieR is on reporting details of the intervention elements (and where relevant, comparison elements) of a study. Other elements and methodological features of studies are covered by other reporting statements and checklists and have not been duplicated as part of the TIDieR checklist. When a **randomised trial** is being reported, the TIDieR checklist should be used in conjunction with the CONSORT statement (see www.consort-statement.org) as an extension of **Item 5 of the CONSORT 2010 Statement.** When a **clinical trial protocol** is being reported, the TIDieR checklist should be used in conjunction with the SPIRIT statement as an extension of **Item 11 of the SPIRIT 2013 Statement** (see www.spirit-statement.org). For alternate study designs, TIDieR can be used in conjunction with the appropriate checklist for that study design (see www.equator-network.org).

For peer review only - http://bmjopen.bmj.com/site/about/guidelines.xhtml
For peer review only

BMJ Open

Supporting adolescents' participation in muscle-strengthening physical activity: Protocol for the 'Resistance Training for Teens' (RT4T) hybrid type III implementation-effectiveness trial

Journal:	*BMJ Open*
Manuscript ID	bmjopen-2023-075488.R2
Article Type:	Protocol
Date Submitted by the Author:	05-Oct-2023
Complete List of Authors:	Kelly, Hayden; The University of Newcastle, School of Education<br>Smith, Jordan ; University of Newcastle, School of Education<br>Verdonschot, Angeliek; The University of Newcastle, School of Education<br>Kennedy, Sarah; Western Sydney University, School of Health Sciences<br>Scott, Joseph; University of the Sunshine Coast; Edith Cowan University, School of Education<br>McKay, Heather; The University of British Columbia, Family Practice<br>Nathan, Nicole; Population Health Hunter New England Local Health District; Hunter Medical Research Institute<br>Sutherland, Rachel; Population Health Hunter New England Local Health District; The University of Newcastle, School of Medicine and Public Health<br>Morgan, Philip; University of Newcastle, PRC for Physical Activity and Nutrition<br>Salmon, Jo; Deakin University,<br>Penney, Dawn; Edith Cowan University, School of Education<br>Boyer, James; NSW Department of Education, School Sport Unit<br>Lloyd, Rhodri S. ; Cardiff Metropolitan University,<br>Oldmeadow, Christopher; Hunter Medical Research Institute<br>Reeves, Penny; University of Newcastle Hunter Medical Research Institute, Health Research Economics; University of Newcastle, School of Medicine and Public Health<br>Pursey, Kirrilly; The University of Newcastle, School of Health<br>Hua, Myna; Western Sydney University Translational Health Research Institute, School of Health Science<br>Longmore, Sarah; Western NSW Local Health District, Health Promotion<br>Norman , Jennifer ; University of Wollongong Illawarra Health and Medical Research Institute; Illawarra Shoalhaven Local Health District, Health Promotion Service<br>Voukelatos, Alexander; University of New South Wales, Population Health Research and Evaluation Hub, Sydney Local Health District<br>Zask, Avigdor; Northern NSW Local Health District, Health Promotion<br>Lubans, David; The University of Newcastle, School of Education
**Primary Subject Heading**:	Global health
Secondary Subject Heading:	Public health, Evidence based practice, Global health

Keywords:	Adolescent, Schools, EDUCATION & TRAINING (see Medical Education & Training)

SCHOLARONE™
Manuscripts

Supporting adolescents' participation in muscle-strengthening physical activity: Protocol for the 'Resistance Training for Teens' (RT4T) hybrid type III implementation-effectiveness trial

Hayden T, Kelly[1], Jordan J, Smith[1,2], Angeliek Verdonschot[1], Sarah G, Kennedy[3], Joseph Scott[4,5], Heather McKay[6], Nicole Nathan[2,7,8,9], Rachel Sutherland[2,7,8,9], Philip J, Morgan[1], Jo Salmon[10], Dawn Penney[11], James Boyer[12], Rhodri S, Lloyd[13], Christopher Oldmeadow[14], Penny Reeves[14], Kirrilly Pursey[15,16], Myna Hua[3], Sarah Longmore[17], Jennifer Norman[18], Alexander Voukelatos[19], Avigdor Zask[20,21], and David R, Lubans[1,2,22,*]

[1] Centre for Active Living and Learning, School of Education, University of Newcastle, Callaghan, New South Wales, Australia

[2] Hunter Medical Research Institute, New Lambton Heights, New South Wales, Australia

[3] School of Health Science, Translational Health Research Institute, Western Sydney University, Kingswood, Australia

[4] University of the Sunshine Coast, Queensland, Australia

[5] School of Education, Edith Cowan University, Perth, Western Australia, Australia

[6] University of British Columbia, Vancouver, Canada

[7] Hunter New England Population Health, Hunter New England Area Health Service, Newcastle Australia

[8] School of Medicine and Public Health, The University of Newcastle, Newcastle, New South Wales, Australia

[9] National Centre of Implementation Science, Newcastle, New South Wales, Australia

[10] Deakin University, Geelong, Victoria, Australia

[11] School of Education, Edith Cowan University, Joondalup, Australia

[12] NSW Department of Education, Sydney, New South Wales, Australia

[13] Youth Physical Development Centre, Cardiff School of Sport and Health Sciences, Cardiff Metropolitan University, Wales

[14] Hunter Medical Research Institute, College of Health, Medicine and Wellbeing, University of Newcastle, New South Wales, Australia

[15] School of Health Sciences, College of Health, Medicine and Wellbeing, University of Newcastle, Callaghan, New South Wales, Australia

For peer review only

For peer review only - http://bmjopen.bmj.com/site/about/guidelines.xhtml

[16] Food and Nutrition Research Group, Hunter Medical Research Institute, New Lambton Heights, New South Wales, Australia

[17] Health Promotion, Western New South Wales Local Health District, Bathurst, New South Wales, Australia

[18] Health Promotion Service, Illawarra Shoalhaven Local Health District, Warrawong, New South Wales, Australia

[19] Population Health Research and Evaluation Hub, Sydney Local Health District, School of Population Health, University of New South Wales, New South Wales Australia

[20] Health Promotion, Northern NSW Local Health District, Lismore

[21] University Centre of Rural Health (Northern Rivers), School of Public Health, University of Sydney, Lismore, NSW Australia

[22] Faculty of Sport and Health Sciences, University of Jyväskylä, Jyväskylä, Finland

Running title

RT4T implementation-effectiveness trial

***Corresponding author**

Professor David Lubans

College of Human and Social Futures, School of Education, University of Newcastle

ATC 303, Advanced Technology Centre

Callaghan, University Drive, NSW, 2308, Australia

Phone: +612 4921 2049

Email: David.Lubans@newcastle.edu.au

Word count: 5338 (excluding authorship statement, reference list and table 1)*

Keywords: School, Resistance training, Implementation, Adolescents, Physical literacy

For peer review only - http://bmjopen.bmj.com/site/about/guidelines.xhtml

ABSTRACT

Introduction: In Australia, only 22% of male and 8% of female adolescents meet the muscle-strengthening physical activity guidelines, and few school-based interventions support participation in resistance training (RT). After promising findings from our effectiveness trial, we conducted a state-wide dissemination of the '*Resistance Training for Teens'* (RT4T) intervention from 2015 to 2020. Despite high estimated reach, we found considerable variability in program delivery and teachers reported numerous barriers to implementation. Supporting schools when they first adopt evidence-based programs may strengthen program fidelity, sustainability, and by extension, program impact. However, the most effective implementation support model for RT4T, is unclear.

Objective: To compare the effects of three implementation support models on the reach (primary outcome), dose delivered, fidelity, sustainability, impact, and cost of RT4T.

Methods and analysis: We will conduct a hybrid type III implementation-effectiveness trial involving Grade 9 and 10 (aged 14 to 16 years) students from 90 secondary schools in New South Wales (NSW), Australia. Schools will be recruited across one cohort (n=90 in 2023, stratified by school type, socio-economic status (SES) and location, and randomised in a 1:1:1 ratio to receive one of the following levels of implementation support: i) 'Low' (training and resources), ii) 'Moderate' (training and resources + external support), or 'High' (training and resources + external support + equipment). Training includes a teacher workshop related to RT4T program content (theory and practical sessions) and the related resources. Additional support will be provided by trained Project Officers (i.e., Project Officers from five local health districts). Equipment will consist of a pack of semi-portable RT equipment (i.e., weighted bars, dumbbells, resistance bands and inverted pull up bar stands) valued at ~AUD$1,000 per school. Study outcomes will be assessed at baseline (T0), 6-months (T1), and 18-months (T2). A range of quantitative (teacher logs, observations, and

For peer review only - http://bmjopen.bmj.com/site/about/guidelines.xhtml

teacher surveys) and qualitative (semi-structured interviews with teachers) methods will be used to assess primary (reach) and secondary outcomes (dose delivered, fidelity, sustainability, impact, and cost of RT4T). Quantitative analyses will utilise logistic mixed models for dichotomous outcomes, and ordinal or linear mixed effects regression models for continuous outcomes, with alpha levels set at $p<.025$ for the outcomes and cost comparisons of the Moderate and High support arms against Low support.

Ethics and dissemination: Ethics approval has been obtained from the University of Newcastle (H-2021-0418), the NSW Department of Education (SERAP:2022215), Hunter New England Human Research Ethics Committee (2023/ETH00052), and the Catholic Schools Office. The trial has been prospectively registered at the Australian Clinical Trials registration (ACTRN12622000861752). The design, conduct and reporting will adhere to the Consolidated Standards of Reporting Trials (CONSORT) statement, the Standards for Reporting Implementation Studies (StaRI) statement, and the Template for Intervention Description and Replication (TIDieR) checklist. Findings will be published in open access peer-reviewed journals, key stakeholders will be provided with a detailed report. We will support on-going dissemination of RT4T in Australian schools via professional learning for teachers.

Strengths and limitations of this study

Strengths and limitation of this study include:

- Our study will test three implementation support models on a range of implementation outcomes and determinants using a hybrid type III implementation-effectiveness trial.
- Local health district staff (i.e., Project Officers) are well placed to support the implementation of school-based interventions, but few studies have examined their capacity to support secondary schools.
- There is potential for secondary school teachers to deliver RT4T with poor fidelity.

For peer review only

- Our study does not include a control condition, but the low support group will serve as a ‘usual practice’ comparator.
- Student-level data will only be collected in a sub-set of schools by secondary teachers, which may compromise internal validity.

For peer review only

For peer review only - http://bmjopen.bmj.com/site/about/guidelines.xhtml

INTRODUCTION

Physical inactivity has been described as a global health issue and is the fourth leading cause of premature death worldwide[1]. The World Health Organisation (WHO) recommends children and adolescents to participate in 60 minutes of (predominantly aerobic) moderate-to-vigorous physical activity (MVPA) daily, as well as muscle strengthening activity at least three days per week[2]. Despite these recommendations, physical activity declines substantially throughout adolescence resulting in many adolescents failing to meet daily physical activity guidelines[3]. Fewer than 1 in 50 Australian adolescents (15 to 17 years old) meet MVPA and muscle strengthening activity recommendations[4]. Poor rates of muscle strengthening activity may explain the secular decline in muscular fitness (i.e., strength, power, and endurance) of Australian youth over the past 30 years[5] with a similar decline seen internationally[6, 7]. This suggests that youth participation in muscle strengthening activity may have declined globally. Muscular fitness is associated with various markers of health and well-being among children and adolescents in both cross-sectional[8] and prospective[9] studies. This prompted our team to investigate the key role of muscle strengthening activity during the school years.

Resistance training (RT) is a specialised form of muscle strengthening activity. It can be performed with or without equipment using a variety of resistive loads, and in a range of settings (e.g., at home, school, local park, or gym/fitness centre)[10]. When performed routinely, RT may lead to muscle hypertrophy (i.e., increase in muscle size), improved muscular fitness (i.e., muscle strength, power, and/or endurance), body composition (i.e., increases in fat free mass and reductions in fat mass), and mental health (i.e., self-esteem) in school-aged youth[11]. RT is also recommended within global physical activity guidelines for adults[1], as it benefits a wide range of physical and mental health outcomes (including risk of chronic disease and depression/anxiety). However, the performance of RT is often

For peer review only

perceived as more complex than aerobic activities (e.g., walking and jogging). This may explain why participation in RT is much lower than for MVPA during adulthood[2]. Gaining the knowledge, skills, and confidence to participate in RT sets children and adolescents up for participation in RT across the lifespan[12]. However, for decades a number of myths and misconceptions about the safety and appropriateness of strength training have prevented it from being offered to most school-aged youth[12]. Furthermore, among adults there are a number of reported barriers to participation in RT, including lack of confidence[13], low self-efficacy[13], lack of time[14], and perceived lack of access to necessary equipment[14]. These barriers may also be present for adolescents, as they likely lack the knowledge, skills and/or confidence to engage in RT despite their desire to try a broader range of physical activities[15]. It is therefore necessary that adolescents are provided with ample opportunities to develop the skills, knowledge, and confidence to engage in RT.

Schools are effective settings for health promotion as they provide access to most children and adolescents, and have the facilities, equipment and qualified staff needed to deliver interventions[16-18]. However, few school-based interventions provided adolescents with the confidence and competence to participate safely in RT[19] and no prior studies have done so at scale[19, 20]. ‘Scale up’ is defined as the ‘deliberate effort to increase the impact of successfully tested health interventions to benefit more people’[21]. Few physical activity interventions have progressed beyond efficacy testing and no studies investigating scaled up RT interventions among secondary students appear within the extant literature[22]. The WHO has advised policy makers, funders, and researchers to focus their efforts on scaling up evidence-based physical activity programs whilst exploring various models of implementation[23]. Prior to investing in scale up, evaluating the effectiveness of models of implementation to inform future scale up is necessary to guide the efficient use of resources. ‘Voltage drop’ is associated with diminishing effectiveness when programs are implemented

For peer review only - http://bmjopen.bmj.com/site/about/guidelines.xhtml

at a larger scale[24, 25]. Evaluating different models of implementation will help to minimise this phenomenon.

Resistance Training for Teens (RT4T) is an evidence-based program designed to provide adolescents with foundational knowledge about RT and enhance competence, confidence, and motivation to engage in RT across the lifespan. It was also designed to align with the Australian Curriculum for Health and Physical Education to develop the movement skills and concepts that will enable students to participate in physical activities that contribute to their health and wellbeing[26]. We conducted a rigorous evaluation of the RT4T intervention using a cluster randomised controlled trial (RCT) in 16 schools (N = 607) between 2015-2016[27]. The intervention resulted in immediate and sustained improvements in upper body muscular fitness and RT skill competency, demonstrating an effective and scalable approach to delivering RT within secondary schools. During this period, we established a partnership with the NSW Department of Education to scale-up the program. With support from the NSW Department of Education, 468 teachers from 249 NSW Government schools were trained to deliver the program[27]. We estimate that the program reached ~10,000 students between August 2015 and October 2020. However, interviews conducted with a sample of teachers (N =19) who delivered RT4T, identified considerable variability in program delivery. Also, several barriers to implementation emerged that included lack of support, motivation and time[28] impacting on program fidelity and potential sustainability.

Implementation support typically used for school physical activity programs, is often criticised as being a ‘train and forget’ model. It comprises initial training workshops followed by limited ongoing support for schools. This approach is unlikely to address the primary impediments to program implementation but may explain some shortcomings in whether evidence-based health promotion programs are adopted, implemented, or maintained within schools. The most effective approach that optimises implementation fidelity and may sustain

For peer review only - http://bmjopen.bmj.com/site/about/guidelines.xhtml

efficacious programs over the longer-term remains unclear[29]. Therefore, the aim of this present trial is to evaluate the effects of three implementation support models on the reach, effectiveness, dose delivered, fidelity, adoption, sustainability, impact, and cost of RT4T.

METHODS

Experimental design

We will conduct a hybrid type III implementation-effectiveness trial design[30] to evaluate the implementation and effectiveness of the RT4T intervention. We aim to recruit 90 secondary schools and randomise them to one of three groups: (i) Low, (ii) Moderate, or (iii) High implementation support. The Low support group will act as a control group (usual practice). This is the professional development model traditionally used by the NSW Department of Education and the delivery model used for RT4T from 2015-2020. In Australia, the academic year is separated into four 'terms' of 10 weeks duration. Following training, teachers enrolled in our study will deliver the 8-week RT4T intervention within one (or more) of two school terms (i.e., within six months post-training). This provides teachers with some flexibility regarding the delivery period for RT4T within their academic year.

Regarding the timeline for the evaluation of the intervention (i.e., the aim of our study), study assessments are undertaken at baseline, 6-months and 18-months follow-up. Teachers will be encouraged to maintain delivering the program after our research has concluded. Our implementation trial is guided by expert recommendations[31] and is registered with the Australian New Zealand Clinical Trials Registry (ACTRN12622000861752). The design, conduct and reporting will adhere to the Consolidated Standards of Reporting Trials (CONSORT)[32] statement, the Standards for Reporting Implementation Studies (StaRI)[33] statement, and the Template for Intervention Description and Replication (TIDieR) checklist[34].

For peer review only - http://bmjopen.bmj.com/site/about/guidelines.xhtml

School recruitment and selection

Secondary schools (Government, Catholic and Independent) in five local health districts in NSW (Illawarra Shoalhaven, Northern NSW, South Eastern Sydney, Sydney, and Western NSW) will be eligible to participate in this study. Schools outside these regions will be able to attend the professional learning workshops and implement RT4T, but will not be included into the study to assess implementation outcomes. Eligible schools will be those that include students in Grades 9 and 10 (aged 14 to 16 years). We will use a range of evidence-based recruitment and retention strategies to maximise participation and minimise drop-out (at both school and teacher levels), including promotion within the NSW Department of Education School Sport Unit newsletter. This will include pre-notification, the use of a dedicated recruitment coordinator, repeated reminders, and deployment of staff from local health promotion teams to engage with schools.

Participants

Teachers: One or two teachers from each enrolled school that teaches students in Grade 9 and 10 will be recruited to act as a 'School Champion'. They will attend a single full-day professional learning workshop delivered by members of the research team with tertiary qualifications in physical education (PE), health promotion, exercise physiology and/or strength and conditioning. School Champions will receive curriculum materials and resources at the workshop and will be asked to deliver a separate 2-hour training for other Grade 9 and 10 teachers at their schools, who also have the option to implement the program in their classes. Teachers can deliver the RT4T sessions to their students during usual PE lessons, co-curricular school sport periods or within an elective subject known as Physical Activity and Sports Studies. Professional learning will align with the NSW Educational Standards

For peer review only - http://bmjopen.bmj.com/site/about/guidelines.xhtml

Authority accreditation process to incentivise teachers to participate. Lastly, the learning will contribute to teachers' required annual training hours on a yearly basis[35, 36].

Students: Secondary students in Grades 9 and 10 (aged 14 to 16 years) enrolled in the study schools will be eligible to participate. The target population has been selected as this is the period during which students start to drop out of organised sport and can benefit from exposure to lifelong physical activities [37] such as resistance training.

Primary and secondary outcomes

A range of quantitative and qualitative methods will be used to assess primary and secondary outcomes at baseline (T0), 6-months (T1) and 18-months follow-up (T2). Our primary outcome is reach, operationalised as the proportion of Grade 9 and 10 students from the study schools who participate in the RT4T program. Proportion will be calculated as a percentage of students from Grade 9 and 10 who participate in ≥50% of the RT4T practical sessions divided by the total number of students in Grade 9 and 10 at the study schools. Secondary outcome measures include, dose delivered, fidelity, sustainability, impact, cost effectiveness, and implementation determinants.

Sample size calculation

We aim to recruit 90 secondary schools, with 30 schools assigned to each of the three treatment arms. This will include an estimated 261 classes and *N*=~7,800 students across the three arms of the trial. Based on our previous research in secondary schools, we estimate 20% of schools will not provide usable data for our primary outcome at follow-up. As few studies have tested the effects of implementation strategies on the reach of the school-based physical activity interventions, there is little information to guide power calculations. Therefore, we estimated that the Low support group will achieve 12.5% reach, while the Moderate and High support groups will both achieve 25% reach (i.e., between group difference of 12.5%). Our

For peer review only - http://bmjopen.bmj.com/site/about/guidelines.xhtml

power calculation is based on 90% power, type 1 error rate of 0.025, and standard deviation of 12%. For the continuous secondary student effectiveness outcomes, we assumed an intraclass correlation coefficient (ICC) estimate of 0.2. If 10% of schools provide useable data (~780 students), our study will have 80% power to detect significant small-to-moderate effects between treatment arms.

Blinding and randomisation

After teachers have completed the RT4T workshop, schools will be randomised to one of three implementation support arms. Schools will be stratified by school type and location and randomised using a random number producing algorithm by an independent statistical analysis service. Data analysis will be conducted by individuals blinded to group allocation.

Evidence-based intervention

RT4T is an 8-week multi-component physical activity program, including practical and theory-based lessons designed to develop adolescents' knowledge, competence, confidence, and motivation to participate in muscle strengthening activity. The design and delivery of RT4T is guided by the Supportive, Active, Autonomous, Fair and Enjoyable (SAAFE) principles for organised physical activity sessions for school-aged youth[38]. The training sessions and progressions were revised based on the Youth Physical Development Centre Basic Resistance Training Curriculum developed by Radnor and colleagues[39].

The RT4T program includes four major components: (i) school-based RT sessions (delivered by teachers); (ii) theoretical classroom sessions (delivered by teachers); (iii) a smartphone phone and tablet app (used by students/ teachers) and (iv) energiser breaks (used by teacher during theoretical sessions).

School-based RT sessions

For peer review only

Teachers will deliver the RT4T program during their usual PE lessons, co-curricular or Physical Activity and Sports Studies. The RT4T practical sessions include four sub-components. Sub-components include: (i) movement based dynamic warm-up (5 minutes), (ii) RT skill development (GymFit: 15-20 minutes) focused on five RT movement categories (i.e., upper body push, upper body pull, lower body bilateral, lower body spilt, and core stability), (iii) choice of three different types of muscle strengthening activities: a fitness workout of the day (WOD), modified game with fitness infusion (GameFit), or a fun fitness challenge done to music (FunFit) (15-20 minutes); and (iv) cool down and static stretching (StretchFit: 5 minutes). Practical sessions will be supported using a variety of resources, including exercise cards (Figure 1) and the RT4T smartphone and tablet app (Figure 2). Teachers will be also provided with a handbook that includes all the practical activities and theoretical content.

Theoretical classroom-based lessons

Students will receive 8 x 40-minute theory-based lessons that accompany weekly practical lessons. Content is purposefully aligned with the Australian curriculum for Health and Physical Education (HPE)[40] and Australian Physical Literacy Framework[41]. The lesson topics are generally relevant to RT and adolescent health. Topics include: (i) introduction to RT, (ii) fitness, self-assessment, and self-monitoring, (iii) fitness and e-Health technologies, (iv) principles of training and RT program design, (v) nutrition and RT: myths and recommendations, (vi) social media, physical activity, and body image, (vii) yoga, Pilates and other community-based muscle strengthening activity; and (vii) reviewing fitness outcomes and realigning future goals.

Smartphone and tablet app

For peer review only - http://bmjopen.bmj.com/site/about/guidelines.xhtml

312 We have developed and updated IOS versions of the RT4T smartphone and tablet app to support teachers to implement the program (see Figure 2). Use of the app is optional and can be downloaded from the Apple app store on all IOS devices and will be utilised for the purpose of tracking fitness test data, providing options for workouts with visual demonstrations for exercise technique along with further resources. The app includes: (i) fitness testing and criterion-referenced standards, (ii) skill testing for evaluating RT skill competence, (iii) workouts pre-designed for random selection of bodyweight RT exercises organised by difficulty using a custom workout generator; and (iv) an exercise library enabling users to browse bodyweight exercises organised by the five RT movement categories.

322 **Implementation strategies that support teachers to deliver RT4T**

323 We will assess whether implementation strategies can be implemented effectively across three study arms that offer implementation support. We define implementation strategies as methods or techniques used to enhance adoption, implementation, or sustainability of the evidence-based intervention[42]. Study arms are (i) Low support or 'usual practice' (training and resources), (ii) Moderate support (training and resources + external support); and (iii) High support (training and resources + external support + equipment) (see Table 1). Implementation strategies were co-developed with our partners in the NSW Department of Education and NSW local health districts and guided by findings from our previous dissemination study[28]. Our strategies align with the taxonomy of strategies as described by Proctor et. al.[42].

333 **Implementation framework and strategies**

334 Our approach is guided by the Consolidated Framework for Implementation Research which describes constructs across five domains: (i) intervention characteristics (RT4T), (ii) outer

For peer review only - http://bmjopen.bmj.com/site/about/guidelines.xhtml

settings (NSW Department of Education), (iii) inner setting (government schools); and (iv) individual (student/teacher) characteristics, and (v) the process of implementation[43]. We describe relevant implementation strategies in Table 1. We categorised implementation strategies as per the Consolidated Framework for Implementation Research constructs and specified their usage using the Action, Actor, Context, Target, Time framework[44].

For peer review only

Table 1: Domains, constructs, and strategies used in our three implementation support models

Domains	Constructs	Strategies	Level of support		
			Low	Moderate	High
**RT4T intervention**	Evidence strength/quality	Findings from the RT4T cluster RCT were used in promotional and training materials.	✓	✓	✓
	Adaptability	Flexible intervention delivery model (PE, school sport or elective) requiring minimal access to equipment.	✓	✓	✓
	Design quality and packaging	Program resources developed by a professional graphic designer.	✓	✓	✓
**Outer setting**	Partnerships and investment	Investment from, and partnership with, key stakeholders from education (i.e., NSW Department of Education) and health (i.e., NSW Health local health districts).	✓	✓	✓
	External policy and incentives	Professional learning accreditation with national and state-based educational standards authorities.<br>Alignment with state school physical activity policy.	✓	✓	✓
**Inner setting**	School culture	School Champions will run a 2-hour face-to-face professional development workshop for other teachers in their school on RT4T. Prior to this, the teachers will have completed a 30-minute pre-learning session on resistance training safety. In the 2-hour workshop the School Champion will:<br>● Present the evidence and the rationale of RT4T.<br>● Teachers will participate in RT4T activities, discuss the content, and gain consensus on how they will deliver RT4T.<br>● The School Champion will share and explain the resources they received at the School Champion workshop.	✓	✓	✓
	Leadership engagement	School Champions and Project Officers will meet with the school principal to ensure commitment. In this meeting they will:<br>● Show the 4-minute promo video which explains the benefits of RT, evidence of RT4T and an overview of the project.<br>● Discuss how RT4T will be delivered in the school, who is involved, the contact person, time commitment and the costs.<br>● Complete the teacher (from all teachers who will deliver RT4T) and principal informed consents and return to the research team by sending to: RTforTeens@newcaste.edu.au		✓	✓
	Resources	Schools will receive exercise technique and workout resources. These will be provided to the School Champion at the teacher workshop. All materials will also be available on a website where teacher can upload all the materials they need to deliver the program.	✓	✓	✓
	Equipment	Schools will be provided with a basic RT equipment pack (~$1,000 AUD). This includes 4x 5kg weighted bars, 4 x 3kg dumbbells, 2 x portable pull up bar stands, and 4 x sets of resistance bands. These will be delivered to the schools in the High support group following randomisation after the workshop.			✓

For peer review only - http://bmjopen.bmj.com/site/about/guidelines.xhtml

**Individuals**	Competence, opportunity, motivation (teacher)	A full-day professional development workshop will be provided to School Champions. This workshop will be accredited with the NSW Educational Standards Authority and counts towards teachers professional learning. This will be delivered by the research team (UoN) who are experts in resistance training and school interventions. These will be held for groups of schools in the local area convenient to their location. Teacher relief for the School Champions will be provided by the School Sport Unit to the school to cover the cost of their attendance. Prior to attending the workshop, School Champions will complete the 30-minute online pre-learning on resistance training safety. The full-day workshop training will cover the following:<br>● Rationale for RT4T (i.e., prevalence of physical inactivity in youth, benefits of RT, and myths/facts regarding participation in RT).<br>● Adaptability of RT4T.<br>● Skills and demonstrations of the movements.<br>● RT4T lesson content and alignment with the Personal Development, Health, and Physical Education syllabus.<br>● Roles and responsibilities of School Champions, teachers, and Project Officers.<br>● Introduction to the RT4T app.	✓	✓	✓
	Perceived barriers (student)	Addresses student motivation using SAAFE principles.<br>● Teachers will learn to deliver RT4T using the SAAFE principles.	✓	✓	✓
**Implementation process**	Planning for implementation	Teachers will complete an action plan template to support implementation of RT4T in their school. The action plan will include:<br>● When they are going to train their staff and how.<br>● When they are going to get resources to teachers and how.<br>● Roles and responsibilities of the School Champion and teachers.<br>● School Champions will complete the action plan during the full-day professional development workshop.	✓	✓	✓
	Project Officers	School Champions will be connected to a Project Officer (i.e., Project Officers from five local health district) who will provide ongoing support to the School Champions after they have attended the workshop. The Project Officer will:<br>● Attend the in person principal meeting with the School Champion to gain principal commitment (only if this is not done before workshop already).<br>● Email/phone/visit the School Champions.<br>● Complete the checklist developed by the research team.<br>● Stay in touch with the project manager from RT4T and provide all the needed details that are requested by the research team (e.g., keep track of the support provided).		✓	✓
	Evaluation and feedback	The Project Officers will observe approximately two RT4T session per school and provide feedback on the RT4T structure via email.<br>● Observations will be conducted using a standardised checklist developed by the research team that assess aspects of the RT4T session (e.g., did the teachers conduct the warm-up?).<br>● Project Officers will only provide feedback on the RT4T structure via email.		✓	✓

345 *Note.* RT4T = Resistance Training for Teens; SAAFE = supportive, active, autonomous, fair, and enjoyable

For peer review only

Professional learning for teachers

The School Champion will complete a professional 30-minute pre-learning online course prior to program delivery. This will include modules covering foundational principles of RT and specific safety recommendations to deliver RT to school-aged youth. Following initial completion of the online learning, the School Champion will attend a single full-day workshop covering the background/rationale, structure, and implementation of the RT4T program, delivered by members of the research team with relevant expertise in physical education, health promotion, exercise physiology, and strength and conditioning.

School Champions will be asked to attend the professional learning workshop and deliver a compressed training module to other Grade 9 and 10 teachers within their schools. The compressed module comprises a train-the-trainer approach that is supported by resources (i.e., presentation slides) provided by the research team. Our goal is to enable all interested and eligible teachers to deliver the RT4T program to maximise the reach of the RT4T program across the eligible student population.

Project Officers

The Project Officers (i.e., Project Officers from five local health districts) will participate in a full-day training delivered by the research team. The training will be focused on the following: (i) RT4T program content, (ii) strategies to support teachers, (iii) Project Officer roles and responsibilities, and (iv) how to assess fidelity of the RT4T program. Following the teacher workshop, schools randomised to the Moderate and High support groups will receive external support from Project Officers). Project Officers with relevant qualifications in physical education, health promotion, exercise physiology, and/or strength and conditioning will observe teachers' implementation of the RT4T practical sessions, provide ongoing support, and address the various barriers to program implementation. School Champions and

For peer review only - http://bmjopen.bmj.com/site/about/guidelines.xhtml

Project Officers in the Moderate and High support groups will meet with school leaders (principal or head of department) to ensure commitment to the RT4T program.

Materials and equipment

All participating schools will receive 100 hardcopy exercise cards to use during practical sessions (see Figure 1). Schools allocated to the High implementation support group will also receive a basic RT equipment pack. Each equipment pack will include a variety of low cost, versatile exercise equipment (e.g., resistance bands,3kg dumbbells and 5kg weighted bar).

Measures and data collection

Quantitative assessments will be conducted at baseline (T0), 6-months (post intervention; T1) and 18-months (1 year post intervention; T2). Teachers will complete an online survey (T0, T1 and T2). Personal Development, Health, and Physical Education Heads of Department (N=18) and School Champions (N=18) will be invited to participate in face-to-face or virtual interviews (T1 and T2).

Implementation evaluation

Primary implementation outcome

We define an implementation outcome as the intentional actions to deliver a policy or an intervention[45, 46].

Reach: Our primary outcome is reach, operationalised as the proportion of Grade 9 and 10 students from the study schools who participate in the RT4T program. Proportion will be calculated as a percentage of students from Grade 9 and 10 who participate in ≥50% of the RT4T practical sessions divided by the total number of students in Grade 9 and 10. We will assess reach by collecting teacher logbooks (i.e., attendance lists) at T1 and T2. We have used logbooks previously in school-based health promotion trials[47, 48] and achieved high

For peer review only - http://bmjopen.bmj.com/site/about/guidelines.xhtml

completion rates, good validity, and reliability[49]. A research team member will request that School Champions provide a copy of teacher logbooks that describe details of the RT4T program.

Secondary implementation outcomes

Representativeness: We will collect representativeness data [(i.e., School Index of Community Socio-Educational Advantage value, indigenous students (%), and students with language backgrounds other than English (%)] at the school level using the My School website. This information will be reported descriptively.

Dose delivered: Dose delivered is the number of RT4T practical and theory lessons delivered to students. Teachers will be asked to record this information in the RT4T teacher handbooks.

Fidelity*:* Project Officers will observe approximately two RT4T practical sessions delivered by a teacher per school (Moderate and High support arms) (total observations N = ~120). The research team will observe approximately one RT4T practical session per school allocated to the Low support arm (total observations N = ~30). Session fidelity will be assessed by completing an observation checklist that describes the RT4T structure and process (e.g., did the teacher include a warm-up?) and to what extent they implemented the SAAFE principles (e.g., teacher was supportive and promoted positive student interactions, with answer options ranging from 1 = '*Strongly disagree*' to 4 = '*Strongly agree*').

Sustainability*:* We define sustainability as the extent to which the program is embedded within school practices. It will be assessed in two ways. First, through an interview with the Personal Development, Health, and Physical Education, Head of Department (at T2). Second, School Champions will be asked to complete the Provider Report of Sustainment Scale (PRESS) sustainability tool[50]. The tool is a brief, pragmatic and generalisable 3-item

For peer review only - http://bmjopen.bmj.com/site/about/guidelines.xhtml

measure for frontline service providers. It assesses evidenced based practice in different settings and has been shown to be a valid measure of sustainment.

Implementation determinants: We define implementation determinants as factors believed or empirically shown to influence implementation outcomes[51]. We will assess the following determinants: culture, acceptability, feasibility, adaptability, compatibility (appropriateness), dose (satisfaction), capabilities, opportunities, and motivations (COM-B) using a teacher survey (~20-minutes). This survey has not been used previously, however is based upon a body of literature per different implementation outcomes including (n = number of survey items): participation in muscle strengthening exercise (6)[52] culture (1)[53], acceptability (1), feasibility (1), compatibility (appropriateness)(1) [54], adaptability (3), dose (satisfaction) (2)[55], capability (4), opportunity (5), and motivation (4)[56]. All School Champions will complete the questionnaire and will advise other teachers delivering the program within their school to do the same. The research team will send reminders for survey completion to all teachers at 6-month and 18-month follow-up.

Impact evaluation

Student-level outcome data will be collected from a convenience sample of students who provide informed parental consent. We anticipate 10% of the sample will provide usable data (N~780 students). Fitness testing, participation in muscle strengthening activity and RT skill competency will be assessed using the RT4T app. The tablet version of the app (adapted from the 'Burn 2 Learn' program)[57] will be used by teachers to assess students' fitness, student's participation in muscle strengthening activity and RT skill competency. We will evaluate upper body muscular endurance using the 90-degree push-up test[58]. Isometric abdominal muscular endurance will be measured using the plank hold test[59]. Standing long jump will assess muscular power, 1-minute sit to stand will assess lower body muscular endurance and

For peer review only

the 10 x 5m shuttle run will assess speed and agility. These fitness assessments are integrated into the RT4T program and conducted by the teachers at the start (week 1) and at the end (week 8) of the 8-week intervention period.

Patient and public involvement: The NSW Department of Health, NSW local health districts, secondary school teachers, and students were involved in the design of the RT4T intervention.

Statistical analysis

Statistical analyses will be conducted by an independent statistical analysis service - Clinical Research Design, Information Technology and Statistical Support from Hunter Medical Research Institute. Analyses of the primary (program reach) and secondary outcomes will be conducted using logistic mixed models (SAS Institute Inc, Cary, NC) for dichotomous outcomes, and ordinal or linear mixed effects regression models for continuous outcomes. The primary outcome (i.e., Reach) will be collected at the school level and assessed using t-tests. Alpha levels will be set at $p<0.025$ for the comparisons of the Moderate and High support arms against the Low support arm. If the p-values for the differences between the Moderate and High support arms with the Low support arm reach this threshold, the Moderate and High support arms will be compared at a 5% significance threshold. For student level outcomes, statistical analyses will be adjusted for the clustering of effects at the class level, as students from each school are nested in classes. Although clustering at the school level is negligible after adjusting for clustering at the class level, we will test this assumption and adjust our analyses for school-level clustering if required. For teacher-level outcomes, clustering will be accounted for at the school-level. Three potential moderators of effects will be explored using interaction terms (i.e., type of Project Officer, socio-economic status of school, and school location). School socio-economic status will be determined using

For peer review only - http://bmjopen.bmj.com/site/about/guidelines.xhtml

the Index of Community Socio-Educational Advantage (ICSEA), which will be obtained from the MySchool website[60]. School ICSEA values are determined using student-level parent occupation and education data, school location (i.e., remoteness), and percentage of indigenous student enrolment.

Data Monitoring

All entered data will be deidentified using participant codes and will be stored electronically within the RT4T app, and in a password protected drive at the University of Newcastle. Data will be checked for implausible values, and 20% of the data will be entered twice to confirm accuracy. It is not expected that participants will be any greater risk of adverse events than they would be when participating in other types of school-based physical activity. However, the teacher handbook includes a section for teachers to report any adverse events that may occur. Any adverse events will be documented and reported to the relevant ethics committee. Any amendments to the study protocols will be publicly available via the Australian and New Zealand Clinical Trials Registry (trial number: ACTRN12622000861752).

Economic evaluation

The economic evaluation will assess the costs and consequences of the RT4T program across the three trial arms and will also include a budget impact analysis. The results of the cost-consequence analysis are presented as a scorecard comprising the total incremental cost of delivering the intervention alongside the range of outcomes reflected in the primary and secondary trial outcomes (consequences). This approach allows decision makers to interpret the costs and outcomes of an intervention in a way that is relevant to their decision-making context.

Costs comprise the resource use associated with the intervention and implementation, while research costs are excluded. The opportunity cost for teacher and staff time will be

For peer review only - http://bmjopen.bmj.com/site/about/guidelines.xhtml

prospectively measured and valued using pro-rata Department of Education salary levels. The implicit cost of the spaces used to conduct the intervention will also be reported, as well as equipment costs. The budget impact analysis will be conducted to estimate the cost of scaling up the intervention across NSW and Australia. Consideration will be given to any cost offsets which would result from the scaling up of the intervention.

ETHICS AND DISSEMINATION

Ethics approval has been obtained from the University of Newcastle (H-2021-0418), the NSW Department of Education (SERAP:2022215), Hunter New England Human Research Ethics Committee (2023/ETH00052), and the Catholic Schools Office. Students attending schools where the program is delivered within the five local health districts will require parental consent for their fitness data to be recorded within the RT4T app. Students attending schools outside the five indicated local health districts will need to provide opt-out consent if they do not want their fitness data collected within the RT4T app. Our findings will be published in open access peer-reviewed journals. We will provide the NSW Department of Education, NSW Ministry of Health, and all participating schools with a detailed report of our study findings. We will support on-going dissemination of RT4T in NSW and beyond via a series of professional learning workshops.

DISCUSSION

Few school-based interventions provide adolescents with the confidence and competence to participate safely in RT and no prior studies have done so at scale[19]. RT4T is an evidence-based program designed to provide adolescents with the competence, confidence, knowledge, and motivation to engage in RT throughout their lifetime. Importantly, RT4T was developed with scale-up in mind using the Consolidated Framework for Implementation Research (CFIR) to guide implementation.

For peer review only - http://bmjopen.bmj.com/site/about/guidelines.xhtml

Our study fills an important gap regarding the scale-up of effective school-based physical activity interventions[61]. Despite the large number of school-based efficacy and effectiveness trials, there is still only a small number of dissemination and implementation trials that have been conducted. As such, little is known regarding the strategies to support the implementation of physical activity programs in schools. While the health benefits are well-established, there is a lack of school-based interventions supporting adolescents' safe participation in structured RT. This research will have widespread population health benefits as we leverage relationships through partnerships with local health district Project Officers and schools to increase program scale-up.

However, there are limitations that should be noted. First, our study does not include a control group. However, the low implementation support group will serve as the RT4T 'usual practice' comparator. Second, our expanded delivery method is reliant upon a 'train-the-trainer' model, and increasing the number of schools involved will help to determine feasibility across varying school contexts. Considering this, there is a risk of compromised fidelity to the intervention. Finally, regarding the effectiveness evaluation, there is a risk of measurement bias influencing internal validity with only a sub-set of schools providing student-level data, which is to be collected by secondary teachers.

Contributors: HTK: software, resources, writing - initial draft, writing - review and editing. JJS: conceptualisation, methodology, software, resources, writing – initial draft, writing - review and editing, supervision and funding acquisition. AV: methodology, software, resources, writing – initial draft, writing – review and editing, supervision, project administration. SGK: conceptualisation, methodology, resources, writing – original draft, writing – review and editing, funding acquisition. JS: conceptualisation, methodology, resources, writing – original draft, writing – review and editing, funding acquisition. HM: conceptualisation, methodology, software, writing – original draft, writing – review and

For peer review only - http://bmjopen.bmj.com/site/about/guidelines.xhtml

editing, funding acquisition. NN: conceptualisation, methodology, writing – original draft, writing – review and editing, funding acquisition. RS: conceptualisation, methodology, writing – original draft, writing – review and editing, funding acquisition. PJM: conceptualisation, methodology, writing – original draft, writing – review and editing, funding acquisition. JS: conceptualisation, methodology, writing – original draft, writing – review and editing, funding acquisition. DP: conceptualisation, methodology, writing – original draft, writing – review and editing, funding acquisition. JB: conceptualisation, methodology, writing – review and editing, funding acquisition. RSL: methodology, resources, writing – review and editing. CO: methodology, writing – review and editing. PR: methodology, review and editing. KP: methodology, resources, writing – review and editing. MH: writing – review and editing, project administration. SL: writing – review and editing, project administration. JN: writing – review and editing, project administration. AV: writing – review and editing, project administration. AZ: writing – review and editing, project administration. DRL: conceptualisation, methodology, software, resources, writing – original draft, writing – review and editing, supervision and funding acquisition.

Funding: This project is funded by a National Health and Medical Research Council (NHMRC) Partnership Grant (APP2010866). DRL is funded an NHMRC Senior Research Fellowship (APP1154507). NN is funded by the Medical Research Future Fund (MRFF) Investigator Grant (GS2000053). This project is supported and co-designed with the NSW Department of Education.

Disclaimer: The study funders will have no role in data collection, analysis, interpretation, or writing nor will they have influence over the publication of findings.

Competing interests: None declared.

Patient consent for publication: Not applicable.

For peer review only

For peer review only - http://bmjopen.bmj.com/site/about/guidelines.xhtml

Provenance and peer review: Not commissioned; externally peer reviewed.

Supplemental material: Not applicable.

Open access: This is an open access article distributed in accordance with the Creative Commons Attribution 4.0 Unported (CC BY 4.0) license, which permits others to copy, redistribute, remix, transform and build upon this work for any purpose, provided the original work is properly cited, a link to the licence is given, and indication of whether changes were made. See: https://creativecommons.org/ licenses/by/4.0/

ORCID IDs:

Hayden T Kelly http://orcid.org/0000-0002-4533-6945

Angeliek Verdonschot https://orcid.org/0000-0002-1519-3402

Sarah Kennedy https://orcid.org/0000-0001-9804-616X

Nicole Nathan https://orcid.org/0000-0002-7726-1714

Philip Morgan https://orcid.org/0000-0002-5632-8529

Jo Salmon https://orcid.org/0000-0002-4734-6354

Dawn Penney https://orcid.org/0000-0002-2000-8953

James Boyer https://orcid.org/0000-0002-0585-4238

Rhodri Lloyd https://orcid.org/0000-0001-8560-1566

Jennifer Norman https://orcid.org/0000-0001-6344-2605

Alexander Voukelatos https://orcid.org/0000-0003-3131-761X

Avigdor Zask https://orcid.org/0000-0002-3888-7335

David R Lubans http://orcid.org/0000-0002-0204-8257

For peer review only

Figure 1. Resistance Training for Teens exercise cards (front and back side)

Figure 2. Resistance Training for Teens app

References

1. Kohl, H.W., et al., *The pandemic of physical inactivity: global action for public health.* The lancet, 2012. **380**(9838): p. 294-305.
2. Bull, F.C., et al., *World Health Organization 2020 guidelines on physical activity and sedentary behaviour.* British journal of sports medicine, 2020. **54**(24): p. 1451-1462.
3. Farooq, M.A., et al., *Timing of the decline in physical activity in childhood and adolescence: Gateshead Millennium Cohort Study.* British journal of sports medicine, 2018. **52**(15): p. 1002-1006.
4. Statistics, A.B.o., *National Health Survey: first results, 2017–18*. 2019, Australian Bureau of Statistics Canberra.
5. Fraser, B.J., et al., *The great leap backward: Changes in the jumping performance of Australian children aged 11− 12-years between 1985 and 2015.* Journal of sports sciences, 2019. **37**(7): p. 748-754.
6. Tomkinson, G.R., et al., *Temporal trends in the standing broad jump performance of 10,940,801 children and adolescents between 1960 and 2017.* Sports medicine, 2021. **51**: p. 531-548.
7. Ortega, F.B., et al., *European fitness landscape for children and adolescents: updated reference values, fitness maps and country rankings based on nearly 8 million test results from 34 countries gathered by the FitBack network.* British journal of sports medicine, 2023. **57**(5): p. 299-310.
8. Smith, J.J., et al., *The health benefits of muscular fitness for children and adolescents: a systematic review and meta-analysis.* Sports medicine, 2014. **44**: p. 1209-1223.
9. García-Hermoso, A., R. Ramírez-Campillo, and M. Izquierdo, *Is muscular fitness associated with future health benefits in children and adolescents? A systematic review and meta-analysis of longitudinal studies.* Sports Medicine, 2019. **49**: p. 1079-1094.
10. Lloyd, R.S., et al., *Position statement on youth resistance training: the 2014 International Consensus.* British journal of sports medicine, 2014. **48**(7): p. 498-505.
11. Brusseau, T., S. Fairclough, and D. Lubans, *The Routledge handbook of youth physical activity*. 2020: Routledge.
12. Faigenbaum, A.D., et al., *Mythology of youth resistance training.* British Journal of Sports Medicine, 2022.
13. Rhodes, R.E., et al., *Factors associated with participation in resistance training: a systematic review.* British Journal of Sports Medicine, 2017. **51**(20): p. 1466-1472.
14. Steele, J., et al., *A higher effort-based paradigm in physical activity and exercise for public health: making the case for a greater emphasis on resistance training.* BMC public health, 2017. **17**(1): p. 1-8.
15. Tannehill, D., et al., *What young people say about physical activity: the Children's Sport Participation and Physical Activity (CSPPA) study.* Sport, Education and Society, 2015. **20**(4): p. 442-462.
16. Hills, A.P., D.R. Dengel, and D.R. Lubans, *Supporting public health priorities: recommendations for physical education and physical activity promotion in schools.* Progress in cardiovascular diseases, 2015. **57**(4): p. 368-374.
17. Poland, B., G. Krupa, and D. McCall, *Settings for health promotion: an analytic framework to guide intervention design and implementation.* Health promotion practice, 2009. **10**(4): p. 505-516.
18. Neufeld, J. and J. Kettner, *The settings approach in public health: thinking about schools in infectious disease prevention and control.* National collaborative center of infectious diseases: University of Manitoba, 2014.

For peer review only - http://bmjopen.bmj.com/site/about/guidelines.xhtml

19. Cox, A., et al., *Efficacy of school-based interventions for improving muscular fitness outcomes in adolescent boys: A systematic review and meta-analysis.* Sports Medicine, 2020. **50**: p. 543-560.
20. Malina, R.M., *Weight training in youth-growth, maturation, and safety: an evidence-based review.* Clinical journal of sport medicine, 2006. **16**(6): p. 478-487.
21. Milat, A.J., et al., *A guide to scaling up population health interventions.* Public health research & practice, 2016.
22. Reis, R.S., et al., *Scaling up physical activity interventions worldwide: stepping up to larger and smarter approaches to get people moving.* The lancet, 2016. **388**(10051): p. 1337-1348.
23. Organization, W.H., *Nine steps for developing a scaling-up strategy*. 2010: World Health Organization.
24. Chambers, D.A., R.E. Glasgow, and K.C. Stange, *The dynamic sustainability framework: addressing the paradox of sustainment amid ongoing change.* Implementation science, 2013. **8**(1): p. 1-11.
25. McCrabb, S., et al., *Scaling-up evidence-based obesity interventions: a systematic review assessing intervention adaptations and effectiveness and quantifying the scale-up penalty.* Obesity Reviews, 2019. **20**(7): p. 964-982.
26. *Rationale*, in *The Australian Curriculum*.
27. Kennedy, S.G., et al., *Implementing resistance training in secondary schools: a cluster randomized controlled trial.* Medicine & Science in Sports & Exercise, 2018. **50**(1): p. 62-72.
28. Kennedy, S.G., et al., *Implementing resistance training in secondary schools: an exploration of teachers' perceptions.* Translational Journal of the American College of Sports Medicine, 2018. **3**(12): p. 85-96.
29. Shoesmith, A., et al., *Barriers and facilitators influencing the sustainment of health behaviour interventions in schools and childcare services: a systematic review.* Implementation Science, 2021. **16**(1): p. 62.
30. Landes, S.J., S.A. McBain, and G.M. Curran, *Reprint of: an introduction to effectiveness-implementation hybrid designs.* Psychiatry research, 2020. **283**: p. 112630.
31. McKay, H., et al., *Implementation and scale-up of physical activity and behavioural nutrition interventions: an evaluation roadmap.* International Journal of Behavioral Nutrition and Physical Activity, 2019. **16**(1): p. 1-12.
32. Health, D.o., *Australia's Long Term National Health Plan.*, C.A. Government, Editor. 2019.
33. Pinnock, H., et al., *Standards for reporting implementation studies (StaRI) statement.* bmj, 2017. **356**.
34. Wolfenden, L., et al., *Obtaining active parental consent for school-based research: a guide for researchers.* Australian and New Zealand journal of public health, 2009. **33**(3): p. 270-275.
35. Kennedy, S.G., et al., *Implementation at-scale of school-based physical activity interventions: A systematic review utilizing the RE-AIM framework.* Obesity Reviews, 2021. **22**(7): p. e13184.
36. Sutherland, R.L., et al., *The physical activity 4 everyone cluster randomized trial: 2-year outcomes of a school physical activity intervention among adolescents.* American journal of preventive medicine, 2016. **51**(2): p. 195-205.
37. Hulteen, R.M., et al., *Development of foundational movement skills: A conceptual model for physical activity across the lifespan.* Sports medicine, 2018. **48**: p. 1533-1540.
38. Lubans, D.R., et al., *Framework for the design and delivery of organized physical activity sessions for children and adolescents: rationale and description of the 'SAAFE' teaching principles.* International journal of behavioral nutrition and physical activity, 2017. **14**(1): p. 1-11.
39. Radnor, J.M., et al., *Developing athletic motor skill competencies in youth.* Strength & Conditioning Journal, 2020. **42**(6): p. 54-70.
40. Australian Curriculum, A.a.R.A., *ACARA - Curriculum*. 2022.
41. Keegan R, B.L., Dudley D., *The Physical Literacy Framework*, S. Australia, Editor. 2019.
42. Proctor, E.K., B.J. Powell, and J.C. McMillen, *Implementation strategies: recommendations for specifying and reporting.* Implementation science, 2013. **8**(1): p. 1-11.

For peer review only - http://bmjopen.bmj.com/site/about/guidelines.xhtml

43. Keith, R.E., et al., *Using the Consolidated Framework for Implementation Research (CFIR) to produce actionable findings: a rapid-cycle evaluation approach to improving implementation.* Implementation Science, 2017. **12**(1): p. 1-12.
44. Presseau, J., et al., *Action, actor, context, target, time (AACTT): a framework for specifying behaviour.* Implementation Science, 2019. **14**: p. 1-13.
45. Proctor, E., et al., *Outcomes for implementation research: conceptual distinctions, measurement challenges, and research agenda.* Administration and policy in mental health and mental health services research, 2011. **38**: p. 65-76.
46. Peters, D.H., et al., *Implementation research: what it is and how to do it.* Bmj, 2013. **347**.
47. Nathan, N., et al., *Multi-strategy intervention increases school implementation and maintenance of a mandatory physical activity policy: outcomes of a cluster randomised controlled trial.* British Journal of Sports Medicine, 2022. **56**(7): p. 385-393.
48. Nathan, N.K., et al., *Implementation of a school physical activity policy improves student physical activity levels: outcomes of a cluster-randomized controlled trial.* Journal of Physical Activity and Health, 2020. **17**(10): p. 1009-1018.
49. Cradock, A.L., et al., *Impact of the Boston active school day policy to promote physical activity among children.* American Journal of Health Promotion, 2014. **28**(3_suppl): p. S54-S64.
50. Moullin, J.C., et al., *Provider REport of Sustainment Scale (PRESS): development and validation of a brief measure of inner context sustainment.* Implementation Science, 2021. **16**(1): p. 1-10.
51. Nilsen, P. and S. Bernhardsson, *Context matters in implementation science: a scoping review of determinant frameworks that describe contextual determinants for implementation outcomes.* BMC health services research, 2019. **19**(1): p. 1-21.
52. Shakespear-Druery, J., et al., *Muscle-Strengthening Exercise Questionnaire (MSEQ): an assessment of concurrent validity and test–retest reliability.* BMJ Open Sport & Exercise Medicine, 2022. **8**(1): p. e001225.
53. Carlson, J.A., et al., *Contextual factors related to implementation of classroom physical activity breaks.* Translational behavioral medicine, 2017. **7**(3): p. 581-592.
54. Weiner, B.J., et al., *Psychometric assessment of three newly developed implementation outcome measures.* Implementation science, 2017. **12**: p. 1-12.
55. Kennedy, S.G., et al., *Process evaluation of a school-based high-intensity interval training program for older adolescents: the burn 2 learn cluster randomised controlled trial.* Children, 2020. **7**(12): p. 299.
56. Keyworth, C., et al., *Acceptability, reliability, and validity of a brief measure of capabilities, opportunities, and motivations ("COM-B").* British journal of health psychology, 2020. **25**(3): p. 474-501.
57. Lubans, D.R., et al., *Time-efficient intervention to improve older adolescents' cardiorespiratory fitness: findings from the 'Burn 2 Learn' cluster randomised controlled trial.* British journal of sports medicine, 2021. **55**(13): p. 751-758.
58. Lubans, D.R., et al., *Test–retest reliability of a battery of field-based health-related fitness measures for adolescents.* Journal of sports sciences, 2011. **29**(7): p. 685-693.
59. Boyer, C., et al., *Feasibility, validity, and reliability of the plank isometric hold as a field-based assessment of torso muscular endurance for children 8–12 years of age.* Pediatric exercise science, 2013. **25**(3): p. 407-422.
60. Australian Curriculum, A.a.R.A. *Guide to understanding the Index of Community Socioeducational Advantage (ICSEA)*. My School 2020; Available from: https://www.myschool.edu.au/media/1820/guide-to-understanding-icsea-values.pdf.
61. Kennedy, S.G., et al., *Implementation and scale-up of school-based physical activity interventions*, in *The routledge handbook of youth physical activity*. 2020, Routledge. p. 438-460.

For peer review only - http://bmjopen.bmj.com/site/about/guidelines.xhtml

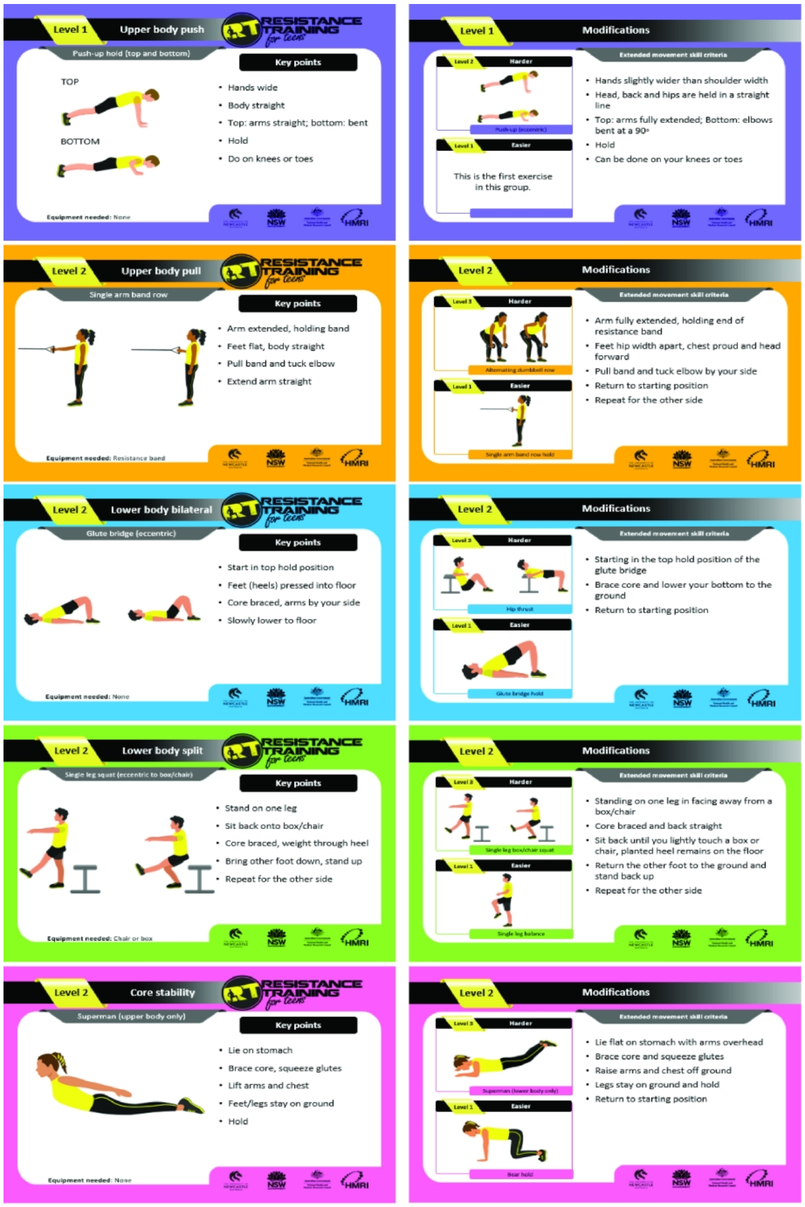

197x297mm (300 x 300 DPI)

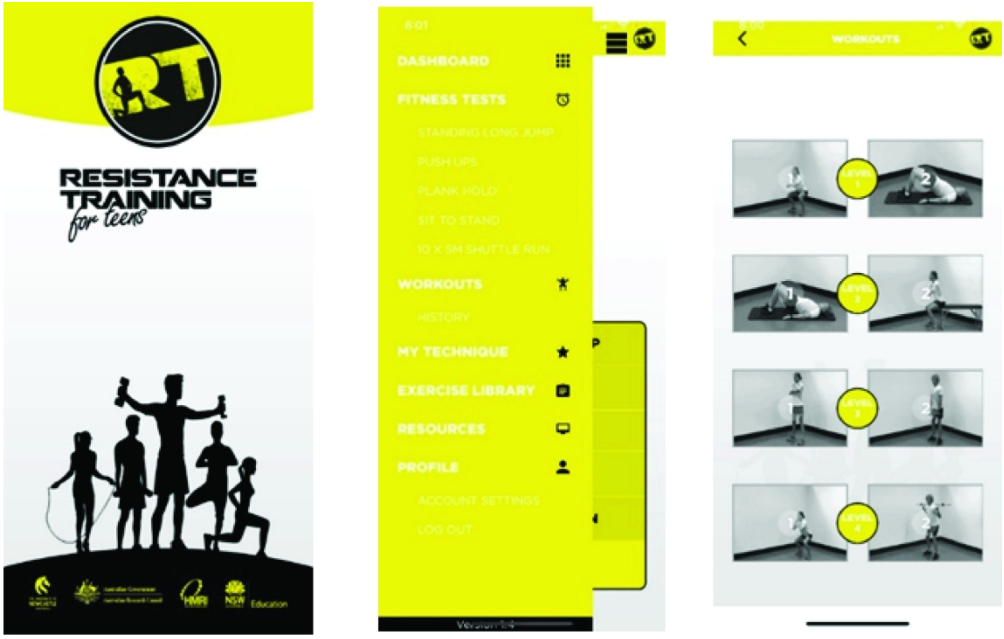

214x135mm (300 x 300 DPI)

TIDieR
Template for Intervention Description and Replication

The TIDieR (Template for Intervention Description and Replication) Checklist*:

Information to include when describing an intervention and the location of the information

Item number	Item	Where located ** Primary paper (page or appendix number)	Other † (details)
	**BRIEF NAME**		
**1.**	Provide the name or a phrase that describes the intervention.	page 2	______________
	**WHY**		
**2.**	Describe any rationale, theory, or goal of the elements essential to the intervention.	pages 7-10	______________
	**WHAT**		
**3.**	Materials: Describe any physical or informational materials used in the intervention, including those provided to participants or used in intervention delivery or in training of intervention providers. Provide information on where the materials can be accessed (e.g. online appendix, URL).	pages 10-11	______________
**4.**	Procedures: Describe each of the procedures, activities, and/or processes used in the intervention, including any enabling or support activities.	pages 10-13	______________
	**WHO PROVIDED**		
**5.**	For each category of intervention provider (e.g. psychologist, nursing assistant), describe their expertise, background and any specific training given.	pages 11,20,21	______________
	**HOW**		
**6.**	Describe the modes of delivery (e.g. face-to-face or by some other mechanism, such as internet or telephone) of the intervention and whether it was provided individually or in a group.	pages 13-14	______________
	**WHERE**		
**7.**	Describe the type(s) of location(s) where the intervention occurred, including any necessary infrastructure or relevant features.	pages 13-14	______________

For peer review only - http://bmjopen.bmj.com/site/about/guidelines.xhtml

	**WHEN and HOW MUCH**		
**8.**	Describe the number of times the intervention was delivered and over what period of time including the number of sessions, their schedule, and their duration, intensity or dose.	pages 13-14	______________
	**TAILORING**		
**9.**	If the intervention was planned to be personalised, titrated or adapted, then describe what, why, when, and how.	pages 13-15	______________
	**MODIFICATIONS**		
**10.‡**	If the intervention was modified during the course of the study, describe the changes (what, why, when, and how).	N/A	______________
	**HOW WELL**		
**11.**	Planned: If intervention adherence or fidelity was assessed, describe how and by whom, and if any strategies were used to maintain or improve fidelity, describe them.	pages 21-22	______________
**12.‡**	Actual: If intervention adherence or fidelity was assessed, describe the extent to which the intervention was delivered as planned.	N/A	______________

** **Authors** - use N/A if an item is not applicable for the intervention being described. **Reviewers** – use '?' if information about the element is not reported/not sufficiently reported.

† If the information is not provided in the primary paper, give details of where this information is available. This may include locations such as a published protocol or other published papers (provide citation details) or a website (provide the URL).
‡ If completing the TIDieR checklist for a protocol, these items are not relevant to the protocol and cannot be described until the study is complete.

* We strongly recommend using this checklist in conjunction with the TIDieR guide (see *BMJ* 2014;348:g1687) which contains an explanation and elaboration for each item.

* The focus of TIDieR is on reporting details of the intervention elements (and where relevant, comparison elements) of a study. Other elements and methodological features of studies are covered by other reporting statements and checklists and have not been duplicated as part of the TIDieR checklist. When a **randomised trial** is being reported, the TIDieR checklist should be used in conjunction with the CONSORT statement (see www.consort-statement.org) as an extension of **Item 5 of the CONSORT 2010 Statement.** When a **clinical trial protocol** is being reported, the TIDieR checklist should be used in conjunction with the SPIRIT statement as an extension of **Item 11 of the SPIRIT 2013 Statement** (see www.spirit-statement.org). For alternate study designs, TIDieR can be used in conjunction with the appropriate checklist for that study design (see www.equator-network.org).

For peer review only - http://bmjopen.bmj.com/site/about/guidelines.xhtml
For peer review only
